# The Influence of Urban Context on Emotions and Bodily Responses During Walking

**DOI:** 10.1007/s11524-025-01051-1

**Published:** 2026-03-27

**Authors:** André Leite Rodrigues, Marta Sofia Aranha da Conceição, Rafael André Luís Ramusga, Carlos Lima Azevedo, Paulo Morgado

**Affiliations:** 1 Associate Laboratory TERRA, Institute of Geography and Spatial Planning, Centre of Geographical Studies, University of Lisbon, Lisbon, Portugal; 2 Institute of Physiology, Lisbon School of Medicine, University of Lisbon, Lisbon, Portugal; 3 Department of Technology, Management and Economics, Technical University of Denmark, Kongens Lyngby, Denmark

**Keywords:** Urban health, Walkability, Physiological response, Environmental stressors, Urban environment

## Abstract

Urban walking offers a unique lens to understand how environmental conditions affect human well-being through both perception and physiological response. This study analyzes 2,207 walking trips from 90 participants from their daily mobility patterns in Lisbon, Portugal, integrating GPS trajectories, physiological signals, self-reported emotions, semantic street imagery, and high-resolution environmental data. We examined how urban features—such as vegetation, noise, sky visibility, infrastructure, and points of interest – relate to peripheral bio signals, like heart rate variability, electrodermal activity, skin temperature, and self-reported emotional states. Results show that moderately green and culturally vibrant environments enhance well-being, calmness, and alertness, while noise, heat, and technical infrastructure increase physiological stress. Clustering analyses reveal that environmental typologies shape affective and bodily responses, with greener and quieter routes yielding more positive outcomes. These findings underscore the embodied nature of walking in cities and highlight the importance of integrating sensory, spatial, and emotional dimensions into urban design for healthier pedestrian experiences.

**Supplementary Information:**

The online version contains supplementary material available at 10.1007/s11524-025-01051-1.

## Introduction

Urban environments influence physical, emotional, and cognitive health through complex interactions between spatial form, environmental exposure, and everyday mobility [1]. Among travel modes, walking is uniquely sensitive to these influences, given its immersive and multi-sensory nature [2, 3]. Research increasingly shows that features such as trees, parks, and human-scale design enhance subjective well-being and reduce stress [4],while noise, heat, and visual chaos are linked to discomfort and physiological arousal [5].

Technological advances now enable real-time tracking of physiological states (e.g., heart rate variability, electrodermal activity) and environmental exposure through GPS, wearables, and volunteered geographic information [6–8]. These tools provide ecologically valid assessments on how urban features influence bodily and emotional states during actual walking episodes, moving beyond simulations or self-reports alone [9, 10].

Several recent studies have expanded this field. Road traffic noise has been shown to modulate psychophysiological responses during forest and urban walks [11]. A Perceived Environment Walking Index (PEWI) was proposed to evaluate route quality [12]. The influence of multi-sensory stimuli on psychological well-being has also been explored [4]. The "Digital Exposome" was introduced to capture real-time environmental and physiological data [13]. In addition, a high-resolution walkability index across Europe was developed [14]. Despite these contributions, many of these studies lack integration between physiological, spatial, and subjective data during real-world movement.

This study addresses that gap by analyzing 2,207 walking trips in Lisbon, Portugal, collected from 90 participants using wearable devices and GPS. Environmental exposure was assessed using high-resolution data on vegetation (NDVI), noise, temperature, visible sky, street-level imagery, and POIs from OpenStreetMap. Subjective perceptions were captured through post-walk questionnaires.

We hypothesize that greener and more vibrant urban environments—characterized by vegetation, visible sky, and the presence of cultural or leisure POIs—are associated with more positive subjective perceptions during walking, such as increased feelings of well-being (H1) [15–19]. Second, we expect that environmental stressors like noise, heat, and technical urban infrastructure are linked to heightened physiological activation, either through increased variability, peak responses, or reduced parasympathetic regulation (H2) [2, 20–23]. Third, we hypothesize that walking routes grouped by their environmental profiles (e.g., greener, noisier) will exhibit significantly different physiological and/or subjective response patterns (H3) [11, 24].

These hypotheses guide the subsequent analyses and inform our examination of how urban form and sensory stimuli influence the embodied experience of walking.

## Methods

### Study Overview

This study was conducted in Lisbon, Portugal—a city with diverse urban morphology suitable for examining how environmental conditions influence pedestrians’ physiological and emotional responses. From August to December 2024, ninety adult residents of the Lisbon Metropolitan Area participated in a two-week monitoring protocol. Participants were recruited via a university mailing list and screened through an online eligibility questionnaire. Selected individuals received detailed written and verbal instructions explaining the use of both devices and daily procedures.

Each participant wore an Empatica E4 wristband continuously to record heart rate, electrodermal activity, and skin temperature, and used the X-ing mobility app to collect GPS trajectories and self-report emotional states after each walking trip using brief semantic differential scales. Surveys were completed directly in the app immediately after each trip. Data were synchronized automatically at the end of each day to a secure server linked to the participant’s account. All procedures were approved by the institutional ethics committee, and participants received a €75 voucher as compensation.

**Fig. 1:**
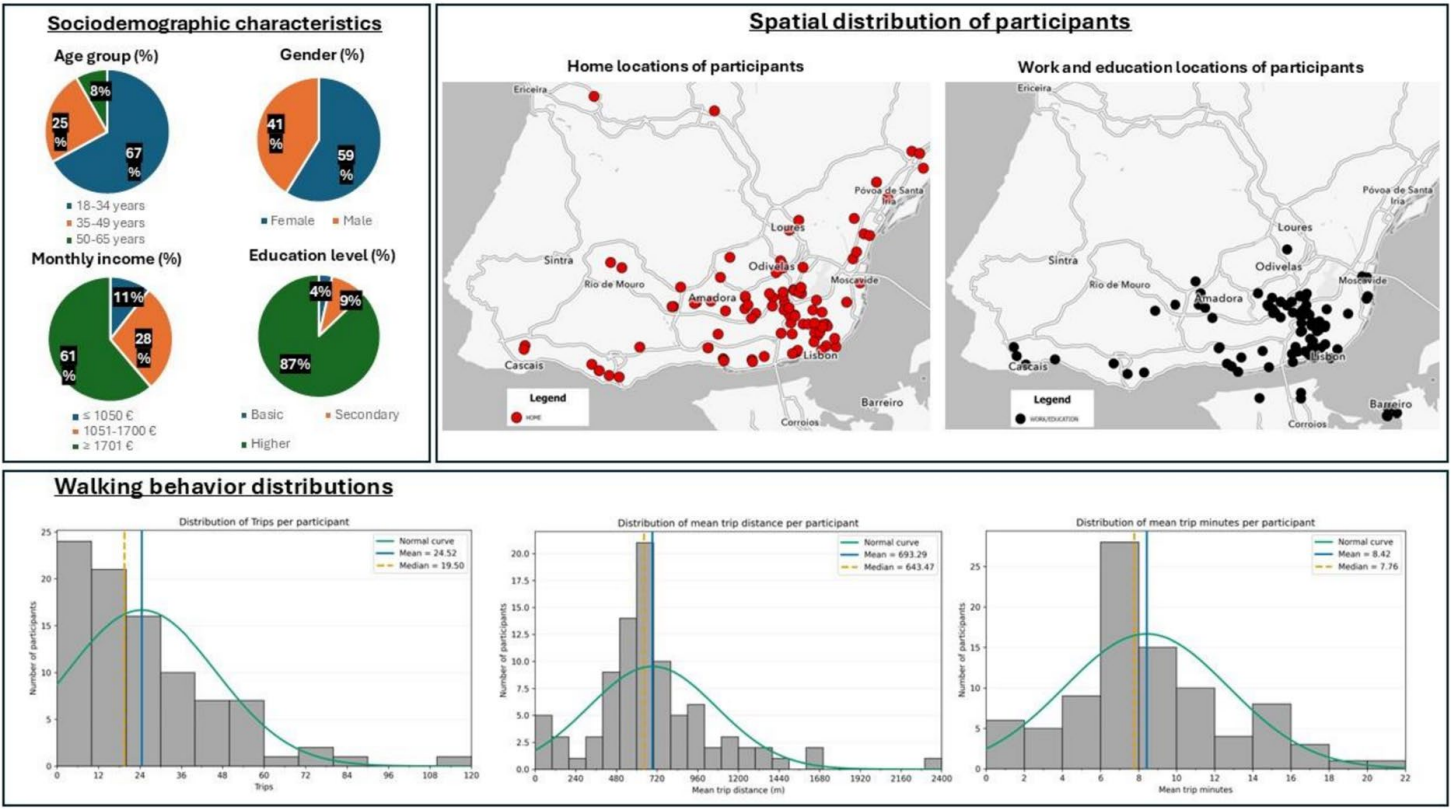
Sociodemographic characteristics, spatial distribution, and walking behavior of participants

In total, 2,207 walking trips were validated and enriched with spatial and environmental indicators, including NDVI, noise, temperature, precipitation, street imagery, and infrastructure variables. Participant characteristics (age, gender, education, income, and residence) and the distribution of daily and total walking trips per participant are presented in Fig. 1and Supplementary Table S6. The methodological workflow (Supplementary Figure S14) builds on a naturalistic mobility protocol previously developed in Copenhagen [25]. The final dataset, including all validated trips and variable descriptions, is publicly available in the repository [26] (*Data_per_route.csv*, *Metadata.csv*).

### Data Preprocessing and Variable Construction

#### GPS Trajectories and Route Construction

GPS data from the X-ing app were used to reconstruct pedestrian routes within Lisbon. The app has been previously applied and validated in mobility research, including pedestrian travel behavior and environmental exposure studies [27, 28]. Points were chronologically ordered by trip ID, and geodesic distances were calculated using the Haversine formula. Points more than 500 m apart were excluded to eliminate signal errors or modal shifts. A simplified stop detection algorithm segmented the data into valid walking trips, removing stationary periods and non-walking segments [29].

Each trip was converted into a LineString, and a 25-m buffer was created to capture micro-scale environmental exposure. This buffer size was chosen to approximate the immediate pedestrian microenvironment in Lisbon, where typical road widths in central neighborhoods range from 10 to 20 m. The 25-m distance, thus, encompasses sidewalks and adjacent elements influencing sensory walking experience, while avoiding dilution from more distant urban features. Comparables buffer sizes (20–30 m) have been employed in previous walkability and environmental exposure studies to represent fine-scale pedestrian environments [1, 30].

Environmental data were extracted from a hexagonal grid (25 m diameter), which ensures uniform cell spacing, minimizes edge effects, and enhances spatial continuity. This resolution effectively captures features like vegetation, street furniture, and infrastructure [31–33].

An overview of the GPS walking trajectories, buffer zones, and spatial coverage of trips across the study area is presented in the Supplementary Figures S1 and S2. These maps illustrate both the extent of the analyzed routes and the variation in trip frequency per cell.

Contextual indicators were derived from Mapillary and POIs from Open Street Map and subsequently aggregated along each route using mean values and frequency distribution across the hexagonal grid.

#### Subjective and Sociodemographic Data

Sociodemographic data were collected via an online pre-survey, including age, gender, education level, and self-reported monthly income [25].

Subjective data were gathered daily through the X-ing app, where participants retrospectively rated four dimensions: (i) Trip Feeling Environment (0–5); (ii) Unwell–Well (1–6); (iii) Agitated–Calm (1–6); and (iv) Tired–Awake (1–6). Participants confirmed the timing and relevance of their responses, increasing ecological validity.

These variables supported the interpretation of physiological responses by capturing emotional and perceptual states during walking trips.

#### Physiological Data Processing

Physiological signals were collected using Empatica E4 wristbands, worn continuously throughout the two-week monitoring period. These devices recorded cardiac activity and skin conductance, allowing the extraction of markers related to stress and autonomic nervous system regulation. Although E4 has been validated for ambulatory monitoring [34, 35] of HRV, electrodermal activity (EDA), and skin temperature, it remains less precise than laboratory-grade systems (e.g., Biopac, Powerlab). Moreover, as data were collected during walking, autonomic variability was naturally constrained by elevated sympathetic tone. To minimize the impact of these limitations, we focused on robust summary measures (e.g., mean RMSSD) and applied standardized preprocessing procedures to enhance signal quality.

From the interbeat interval (IBI) data, we computed heart rate variability (HRV) metrics: RMSSD (short-term parasympathetic activity), pNN50 (percentage of successive IBI differences > 50 ms), and mean heart rate (bpm) as a general cardiac marker. HRV indices were derived using 10-s windows by applying standard formulas to all IBIs within each interval and then summarizing per walking trip (mean and dispersion measures).

Electrodermal activity (EDA) was first smoothed with a Savitzky–Golay filter, after which tonic and phasic components were separated using a high-pass filter. As the phasic component reflects rapid changes in response to environmental stimuli, we derived features including skin conductance response amplitude, rise time (onset to peak), and recovery time (time to return to 63% of peak). For these EDA-derived metrics, mean values and standard deviations were computed at the trip level. To illustrate the signal quality and preprocessing steps, examples of raw and filtered EDA waveforms are provided in Supplementary Figures S13a–b.

To ensure signal validity, only sessions lasting ≥ 2 min, containing non-empty IBI arrays, and showing evidence of proper sensor contact (skin temperature ≥ 30 °C or raw EDA ≥ 0.05 µS) were retained for analysis. Trips with poor signal quality were excluded.

All physiological indicators were computed per walking trip and synchronized with GPS trajectories. Trips with poor signal quality were excluded.

Data processing followed the Emotional Cities experimental protocol [36, 37].

#### Climatic Data Acquisition and Processing

Daily temperature and precipitation values were obtained from the Portuguese Institute of the Sea and Atmosphere (IPMA) [38]. Maximum, mean, and minimum temperatures, as well as daily precipitation totals, were matched to each walking trip based on date, meaning that climatic variation reflects temporal variation between days, not spatial variation within the city. These variables were included as covariates to account for weather effects. Full methodological details are available in Supplementary Methods S2.4.

#### NDVI Extraction and Processing

Vegetation exposure was measured using NDVI composites from Sentinel-2 imagery processed in Google Earth Engine for August–December 2024. Monthly cloud-filtered mosaics (10 m) were clipped to Lisbon and summarized per walking route using mean, min, max, and SD values within the 25 m buffer. NDVI metrics were then used as continuous predictors in subsequent models (details in Supplementary Methods S2.5).

#### Noise Pollution Data Processing

Environmental noise exposure was derived from Lisbon’s official Lden noise map (dB[A]), provided by the municipality [39]. Values were extracted for each walking trip by intersecting noise rasters with 25 m buffers around GPS tracks, generating mean, minimum, maximum, and SD noise metrics per route. These indicators were included as continuous predictors of perceptual and physiological responses (full processing details in Supplementary Methods S2.6).

#### Streetscape Feature Extraction from Mapillary Imagery

Streetscape characteristics were derived from Mapillary street-level imagery using semantic segmentation applied to geolocated photographs intersecting a 25-m buffer around each route. Images were classified into visual categories (vegetation, sky, road, sidewalks, vehicles, poles, etc.) using a pretrained deep-learning model, and the mean pixel-proportion per class was computed to quantify visual exposure along each trip (full pipeline in Supplementary Methods S2.7) [40, 41].

#### Points of Interest (POI) from OpenStreetMap

Points of Interest (POIs) from OpenStreetMap were used as indicators of urban functional diversity, including services, commerce, tourism, public facilities, and greenery. POIs were extracted via the Overpass API and aggregated within a 25-m buffer around each walking route. Counts per category were summed to represent exposure intensity along each trip, and these indicators were later incorporated into statistical analyses [41].

Full data processing workflow, category definitions, and extraction scripts are documented in Supplementary Methods S2.8.

### Statistical Analysis

The unit of analysis was the individual walking trip, with multiple trips nested within participants. Environmental and infrastructural predictors were continuously monitored along each route and aggregated at the trip level to create summary statistics (mean, maximum, and standard deviation) within a 25 m buffer around each GPS trajectory. These trip-level summaries were used as predictors in the models.

To account for repeated measures and the nested data structure, linear mixed-effects models with random intercepts for participant ID were fitted. Each model included multiple environmental predictors simultaneously, allowing estimation of their independent associations with perceptual and physiological outcomes while accounting for correlations among them. Pearson correlations were computed to explore bivariate relationships and guide model specification by minimizing multicollinearity (see Supplementary Tables S1– S3). Person-level characteristics (age, gender, education, income) were collected but not included as fixed effects, as the sample was relatively homogeneous and the study focused on within-individual variation across environmental contexts.

Hypothesis 1 (H1) posited that greener and more vibrant environments are associated with more positive perceptions. Separate mixed-effects models were estimated for four subjective outcomes (*Trip Feeling Environment*, *Unwell–Well*, *Agitated–Calm*, *Tired–Awake*), with standardized environmental indicators (NDVI, Mapillary-derived vegetation and sky visibility, sidewalk presence) and POI densities (leisure, tourism, arts, greenery, water bodies, slope) entered jointly as predictors. Missing POI values were replaced with zero. Models were implemented in Python’s MixedLM function (*statsmodels*), with α = 0.05. Full specifications and preprocessing steps are detailed in “6.Model_H1.py” (Zenodo repository [26]).

Hypothesis 2 (H2) examined whether environmental stressors (noise, heat, infrastructure) are associated with increased physiological activation. Using the same framework, predictors were grouped into five thematic blocks (Noise, Temperature, Technical Infrastructure, Functional POIs, Visual Elements), and each block was tested in separate models across 27 physiological indicators of HRV, EDA, and skin temperature. All predictors were standardized before analysis. Associations meeting both statistical significance (p < 0.05) and effect-size criteria (|β|> 0.1) were retained. Full details are available in “7.Model_H2.py” (Zenodo repository [26]).

Hypothesis 3 (H3) evaluated whether distinct environmental typologies elicit differentiated physiological and perceptual responses. K-means clustering was applied to standardized environmental variables (NDVI, sky visibility, vegetation, noise, and POI categories), with missing POIs imputed as zero. The optimal number of clusters (k = 4) was determined using the elbow method, producing typologies representing distinct urban environments (e.g., green/quiet vs. dense/noisy). Physiological and perceptual outcomes were compared across clusters using Kruskal–Wallis tests. Details of the clustering workflow and comparison methods are provided in “8.Model_H3.py” (Zenodo repository [26]).

All analyses were conducted in Python (Pandas, Statsmodels, Scikit-learn), with spatial preprocessing in ArcGIS Pro and data cleaning in MS Access. Statistical significance was set at α = 0.05, with adjustments for multiple comparisons when appropriate. This analytical framework enabled both explanatory and exploratory evaluation of how urban environmental typologies shape emotional and physiological responses during walking.

## Results

### Subjective Perceptions and Environmental Attributes (H1)

Self-reported perceptions after each walking trip showed identifiable and consistent relationships with environmental conditions along routes. Bivariate correlations (Supplementary Table S1) indicated that more culturally active and greener areas were generally perceived as more pleasant — for instance, Trip Feeling Environment correlated positively with tourism POIs (r = 0.25), leisure (r = 0.21), and natural greenery (r = 0.12). Calmness also increased with leisure (r = 0.30) and tourism (r = 0.25), suggesting that engaging and socially vibrant urban spaces promote emotional relaxation.

The mixed-effects regression models corroborated these results while adjusting for other predictors (see Table 1). Curb presence emerged as the most consistent positive predictor of perception, increasing Unwell–Well (β = 0.078, p = 0.046), Agitated–Calm (β = 0.092, p = 0.036), and Tired–Awake (β = 0.099, p = 0.042). Interpreted practically, this means that a one–standard-deviation increase in curb exposure corresponds to an improvement of approximately + 0.08 to + 0.10 units in reported well-being — detectable but modest uplift on the 0–5 scale.

**Table 1:** Key Environmental Predictors of Self-Reported Feelings During Walking (H1)

Environmental predictor	Unwell– Well (β)	Agitated– Calm (β)	Tired– Awake (β)	Interpretation
Curb presence	0.078	0.092	0.099	Curbs consistently improve perceived well-being, calmness and alertness
NDVI mean	—	0.126	0.129	Greener routes feel calmer and more energizing
NDVI max	—	–0.094	—	Very dense vegetation slightly reduces calmness
Sky visibility	—	–0.115	—	Excessive openness decreases calmness
Water bodies	–0.073	—	—	Water presence slightly lowers well-being
Tourism POIs	—	—	0.127	Cultural destinations increase alertness

*p < 0.05

Effects of greenery were similarly directional but more nuanced. Mean NDVI increased calmness and vitality (≈ + 0.12–0.13 per 1-SD), while maximum NDVI and sky openness predicted slight decreases in calmness (β ≈ –0.09 to –0.12), indicating that dense vegetation or excessive openness may reduce perceived comfort under some conditions. Tourism POIs also increased alertness (β = 0.127 for Tired–Awake, p = 0.006), suggesting that cultural destinations contribute to heightened attentiveness during walking.

Coefficients (β) represent adjusted mean differences in perception scores associated with a one-standard-deviation increase in each predictor (linear mixed-effects models, trip-level; random intercept = participant). Only significant effects (p < 0.05) shown. Full model outputs in Supplementary Table S2.

### Environmental Stressors and Physiological Responses (H2)

Hypothesis 2 proposed that environmental stressors—such as noise, heat, and infrastructure—would increase physiological activation during walking, reflected in EDA and HRV indicators. Correlation analyses (Supplementary Table S3) partially supported this expectation. Heat exposure emerged as the most consistent stressor: higher ambient temperatures were associated with increased EDA mean amplitude (r = 0.23) and reduced RMSSD (r = –0.13), indicating stronger sympathetic activation and lower vagal regulation. Noise showed weaker correlations with EDA amplitude but modest positive associations with rise-time features, suggesting more subtle electrodermal reactivity.

Mixed-effects models (Table 2; Supplementary Table S4) further confirmed that heat and noise were the strongest predictors of physiological stress. Regression coefficients (β) reflect the adjusted mean difference in each physiological outcome associated with a one–standard-deviation increase in the standardized environmental predictors (z-score), accounting for repeated trips nested within participants. Higher maximum temperatures were linked to increased EDA mean amplitude (β = 0.16, p = 0.0002) and slightly higher mean RMSSD (β = 0.087, p = 0.018), consistent with enhanced sympathetic mobilization and reduced parasympathetic control. Similarly, noise exposure predicted stronger EDA activation (β = 0.18, p = 0.0002).

**Table 2:** Key Environmental Predictors of Physiological Stress Responses During Walking (H2)

Predictor (Exposure)	Outcome (Physiology)	β (mean difference)	*p*-value	Interpretation
Temperature	↑ EDA mean amplitude	0.16	0.0002	Higher temperature increases sympathetic arousal
	↑ RMSSD	0.087	0.018	Slight increase in vagal-related HRV under heat load
Noise	↑ EDA mean amplitude	0.18	0.0002	Noise elevates electrodermal activation
Infrastructure – Curbs	↑ LF/HF ratio	0.058	0.0013	More curbs correspond to sympathetic dominance
Infrastructure – Poles	↓ LF/HF ratio	–0.030	0.028	Poles associated with reduced stress balance
Visual exposure – Cars	↑ Mean BPM	2.19	0.0012	Traffic presence increases cardiovascular activation

Built environment features also modulated autonomic responses. Curbs were associated with higher LF/HF ratios (β = 0.058, p = 0.0013), signaling sympathetic dominance, while poles showed the opposite effect (β = –0.030, p = 0.028). Visual cues reinforced this pattern: the presence of cars predicted higher mean heart rate (β = 2.19, p = 0.0012). Overall, these results confirm that heat, noise, and infrastructural elements elevate physiological strain, whereas social and visual cues did not exert a measurable buffering effect on cardiac variability during walking.

β coefficients represent the mean difference in each physiological outcome associated with a 1-SD increase in the standardized environmental predictor (all predictors z-scored). Models include random intercepts for participant ID to account for repeated trips. Full model outputs are reported in Supplementary Table S4.

### Environmental Clusters and Psychophysiological Variation (H3)

K-means clustering applied to standardized environmental variables (greenery, noise, infrastructure, and POIs) identified four distinct urban typologies (k = 4; see Supplementary Figure S8). Cluster 0 represented low-density areas with sparse vegetation and minimal noise; Cluster 1 featured abundant greenery and openness with moderate functional diversity; Cluster 2 combined moderate greenery with mixed-use urban layouts; and Cluster 3 encompassed dense infrastructure, high commercial activity, and elevated noise exposure. Key psychophysiological differences between clusters are presented in Table 3, with full test outputs listed in Supplementary Table S5.

**Table 3:** Differences in Physiological and Subjective Outcomes Across Environmental Walking Clusters (H3)

Outcome domain	Variable	Kruskal– Wallis χ^2^	p-value	Interpretation
Electrodermal activity (EDA)	Mean amplitude	12.24	0.0066	Higher in dense/noisy (Cluster 3) → increased sympathetic activation
	Min amplitude	18.84	0.0003	Strong activation in high-density urban environments
	STD amplitude	8.09	0.0441	Greater variability in EDA in stressful contexts
	Mean rise-time (npoints)	14.26	0.0026	Faster reactivity peaks under stressor-rich conditions
Skin temperature	STD values	17.77	0.0005	Larger thermal fluctuations under environmental load
	Min values	11.55	0.0091	Lower peripheral temperature in Cluster 3 → vasoconstriction
Subjective perception	Trip Feeling Environment	9.77	0.0207	Clusters 0–1 rated more pleasant vs. Cluster 3

Kruskal–Wallis tests demonstrated clear psychophysiological variation across clusters (Table 3; full outputs in Supplementary Table S5). For subjective perceptions, Trip Feeling Environment differed significantly between groups (χ^2^ = 9.77, p = 0.021), with more pleasant evaluations in the greener and quieter Clusters 0 and 1, and less positive affect in the dense, noisy Cluster 3.

Physiological patterns aligned with these perceptual trends. EDA reactivity—mean amplitude (χ^2^ = 12.24, p = 0.0066), minimum amplitude (χ^2^ = 18.84, p = 0.0003) and rise time (χ^2^ = 14.26, p = 0.0026)—was highest in Cluster 3, indicating stronger sympathetic arousal under infrastructural density and acoustic load. Skin temperature deviation (χ^2^ = 17.77, p = 0.0005) and minimum skin temperature (χ^2^ = 11.55, p = 0.0091) were lowest in the same cluster, suggesting thermoregulatory stress. Although heart rate and HRV outcomes did not reach significance, trends in minimum bpm (χ^2^ = 6.62, p = 0.085) and LF/HF ratio (χ^2^ = 6.71, p = 0.082) pointed toward sympathetic dominance in dense urban contexts.

**Fig. 2:**
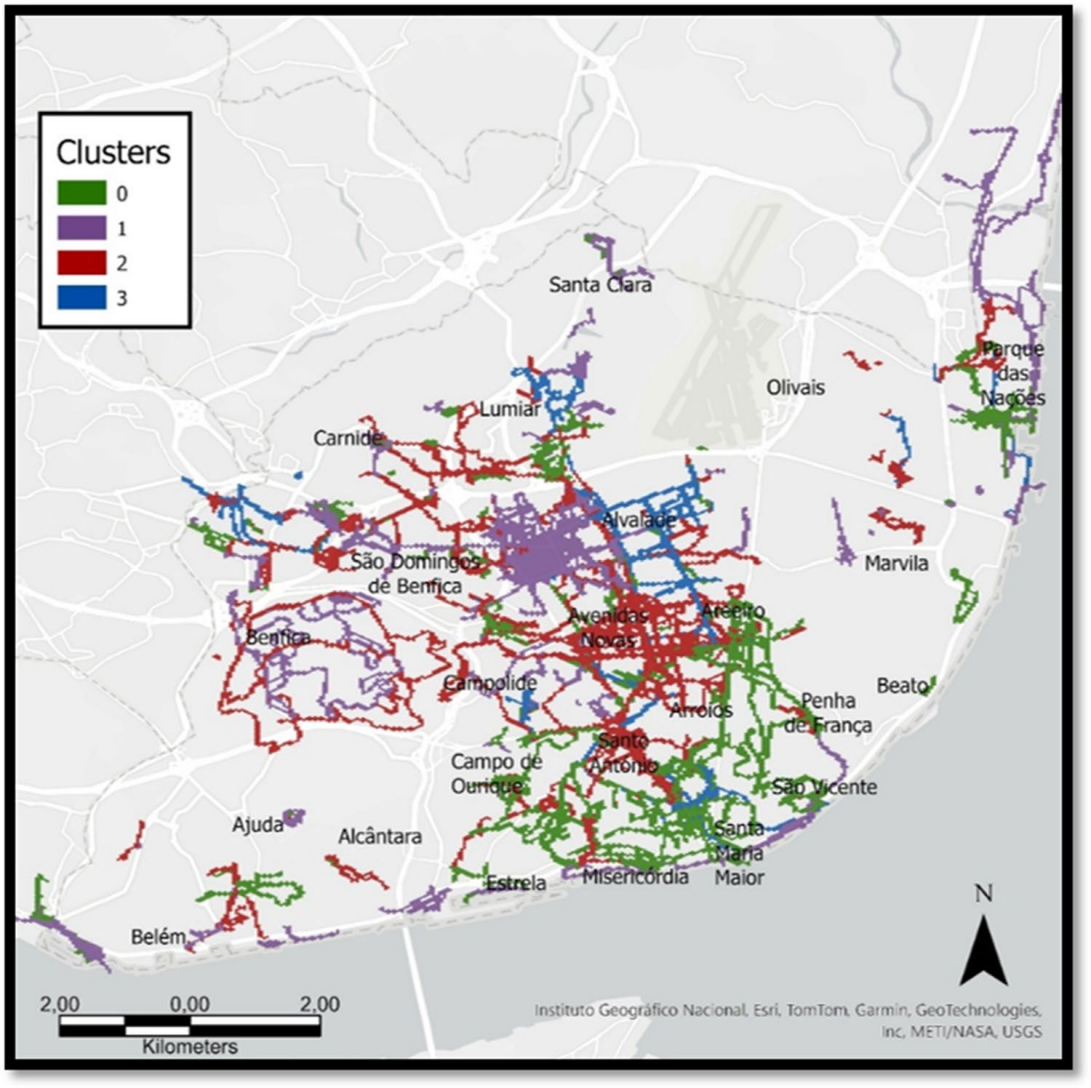
Clustered Pedestrian Routes Across Lisbon Based on Environmental Characteristics

Overall, Cluster 3 consistently showed heightened autonomic activation and less favorable subjective experience, whereas Clusters 0–1 aligned with calmer and more positive responses. The spatial distribution of clusters is displayed in Fig. 2, with representative street-level imagery shown in Supplementary Figures S9– S12.

Kruskal–Wallis statistics (χ^2^) represent differences in physiological and perceptual outcomes between environmental clusters derived via K-means classification (based on NDVI, noise, POIs and Mapillary features). Higher EDA and skin temperature values indicate stronger sympathetic activation. Only significant results (p < 0.05) are shown; full distributions including non-significant outcomes are reported in Supplementary Table S5.

## Discussions

### Subjective Perceptions and Environmental Attributes (H1)

These findings suggest that subjective responses during walking are not uniformly driven by greenery or openness alone, but rather by an interplay of environmental and cultural factors. While many bivariate correlations were modest, the multivariate models revealed clearer relationships once covariates were controlled. NDVI, for instance, showed selective effects, with mean NDVI associated with increased calmness and energetic states, whereas maximum NDVI demonstrated a small negative relationship with calmness.

The positive influence of moderate vegetation aligns with prior studies emphasizing the perceptual and emotional benefits of greenery, whereas excessive openness or dense vegetation may reduce visibility and increase perceived insecurity [15, 17]. The consistent effects of curbs support previous findings on the role of micro-infrastructure in promoting walkability and emotional comfort [16].

Tourism-related POIs were associated specifically with higher perceived energy during walking, reinforcing the notion that urban vibrancy enhances walking experiences [18, 19]. Conversely, the negative association between water bodies and well-being challenges conventional assumptions about their restorative potential, possibly reflecting fears related to entrapment or inadequate lighting [17].

Overall, Hypothesis 1 was only partially supported. Positive pedestrian perceptions emerged from a balanced combination of vegetation, visibility, infrastructure, and cultural vitality rather than from greenness alone. This highlights the need for multidimensional design strategies in urban environments.

### Environmental Stressors and Physiological Responses (H2)

These findings indicate that noise and heat consistently increase physiological activation, supporting Hypothesis 2. The associations between maximum noise and EDA measures suggest heightened autonomic arousal, which is consistent with previous studies [23].

Thermal effects mirrored prior evidence that heat suppresses vagal activity and amplifies electrodermal responses [20]. The observed influence of visual and social stimuli (e.g., cars, pedestrians) further suggests that everyday urban encounters can modulate cognitive load and stress responses, as reported in previous studies [2, 22].

The mixed associations with micro-scale infrastructure (e.g., poles, curbs) highlight their nuanced role in shaping physiological stress. While some features dampened variability, others amplified it, reflecting the complex interplay between attention, perceived safety, and environmental cues.

Overall, these results reinforce the importance of designing urban environments that mitigate auditory and thermal stressors while carefully managing visual and infrastructural stimuli to foster calmer, healthier walking experiences.

### Environmental Clusters and Psychophysiological Variation (H3)

These findings support Hypothesis 3, indicating that combined environmental characteristics jointly shape both perception and physiological reactivity during walking. Participants exposed to greener, quieter environments (Clusters 0 and 1) reported more positive emotional states. In contrast, Cluster 3, characterized by dense infrastructure, commercial intensity, and higher modeled noise, showed significantly higher EDA amplitude and reduced skin temperature stability, reflecting increased sympathetic activation and reduced thermal equilibrium.

This pattern aligns with evidence that traffic noise undermines walking’s restorative potential [11], and that dense, stimulus-rich environments elevate arousal responses [24]. Similarly, prior studies demonstrate that natural settings support emotional regulation and stress recovery during urban movement [15], consistent with the more favorable affective evaluations observed in greener clusters. Although HR and HRV indicators did not differ significantly across clusters, small directional trends suggested greater autonomic tension in Cluster 3 relative to Clusters 0–1.

Overall, these results reinforce that environmental context meaningfully shapes both emotional experience and bodily stress responses during real-world walking. Cluster-based typologies therefore provide an ecologically grounded way to interpret how sensory, structural, and functional features of urban form condition pedestrian well-being.

### Limitations

This study presents a high-resolution, ecologically valid dataset combining physiological, spatial, and perceptual data from over 2,000 real-world walking trips in Lisbon. Despite its strengths, some limitations should be noted. As walking routes were self-selected, environmental exposure was not randomized, restricting causal inference. Unobserved factors such as mood, physical condition, or route familiarity may have influenced responses. Physiological data from wearable devices (Empatica E4), though validated for ambulatory use, are less precise than laboratory-grade systems, and walking conditions inherently constrain autonomic variability, increasing susceptibility to artifacts. Although quality control procedures were rigorous, some residual noise may remain; however, the large sample, repeated measures, and convergence of subjective and physiological indicators strengthen confidence in the results.

Environmental data also posed constraints: while NDVI and weather were time-matched, other layers (Mapillary, noise maps, POIs) were static and may not fully reflect real-time conditions. Coverage gaps or inconsistencies in Mapillary and OpenStreetMap could lead to uneven representation of urban features. Automated image classification and geospatial overlays, though detailed, do not capture subjective perceptions of place and may include segmentation errors or spatial duplications. Finally, the analysis did not account for social context indicators such as deprivation or inequality, which may influence environmental exposures and health outcomes. Future research integrating socioeconomic data could improve explanatory power. Despite these limitations, the convergence between physiological and self-reported responses across environmental clusters supports the robustness and ecological validity of the findings.

### Implications and Future Research

Our findings highlight that urban environmental quality is multidimensional: greenery alone does not ensure well-being unless balanced with visibility, infrastructure, and cultural vitality. Small-scale features such as sidewalks and pedestrian amenities consistently predict comfort, while noise and heat act as stressors, emphasizing the need for interventions that reduce sensory overload. Environmental clustering offers a practical tool for planners to identify areas where walkability and well-being are at risk. Future research should integrate social environment variables (e.g., poverty, housing, inequality) to capture contextual influences, and replicate analyses across diverse urban settings to enhance generalizability. Methodological improvements—such as higher-precision physiological sensors, dynamic environmental data, and qualitative approaches like interviews or ecological momentary assessments—could deepen understanding of how environmental exposure shapes the physiological and emotional dimensions of walking.

## Conclusion

This study advances understanding of how urban environments shape pedestrian well-being by integrating physiological signals, spatial indicators, and subjective perceptions from over 2,200 walking trips in Lisbon. The findings show that moderately green, culturally active areas—combining vegetation, tourism amenities, and pedestrian infrastructure—are linked to greater well-being, calmness, and vitality, while densely vegetated or overly open areas evoke more complex or negative perceptions, highlighting the context-dependent nature of restorative experiences. Physiological results indicate that noise, heat, and dense infrastructure heighten sympathetic activation and thermal stress, whereas greener, quieter spaces promote more favorable autonomic patterns. These outcomes underscore the multisensory and embodied character of urban walking and suggest that urban design should adopt a holistic perspective integrating environmental complexity, sensory experience, and emotional comfort. Despite some limitations, the study provides ecologically valid evidence with practical implications for creating healthier, more inclusive, and emotionally supportive urban environments.

## Supplementary Information

Below is the link to the electronic supplementary material.

ESM 1 (PDF 4.42 MB)

ESM 2 (PDF 204 KB)

ESM 3 (PDF 458 KB)

## Data Availability

The full dataset, variable descriptions, and analysis scripts are publicly available at Zenodo: 10.5281/zenodo.16092877.
