# Supplementary material for: The Influence of Urban Context on Emotions and Bodily Responses During Walking
Source: J Urban Health. 2026 Mar 27;103(2):344–56. doi: 10.1007/s11524-025-01051-1 (PMC13235672; doi:10.1007/s11524-025-01051-1)
Supplement: Supplementary file 1 — (PDF 4.42 MB) [file 11524_2025_1051_MOESM1_ESM.pdf]

Supplementary Figures

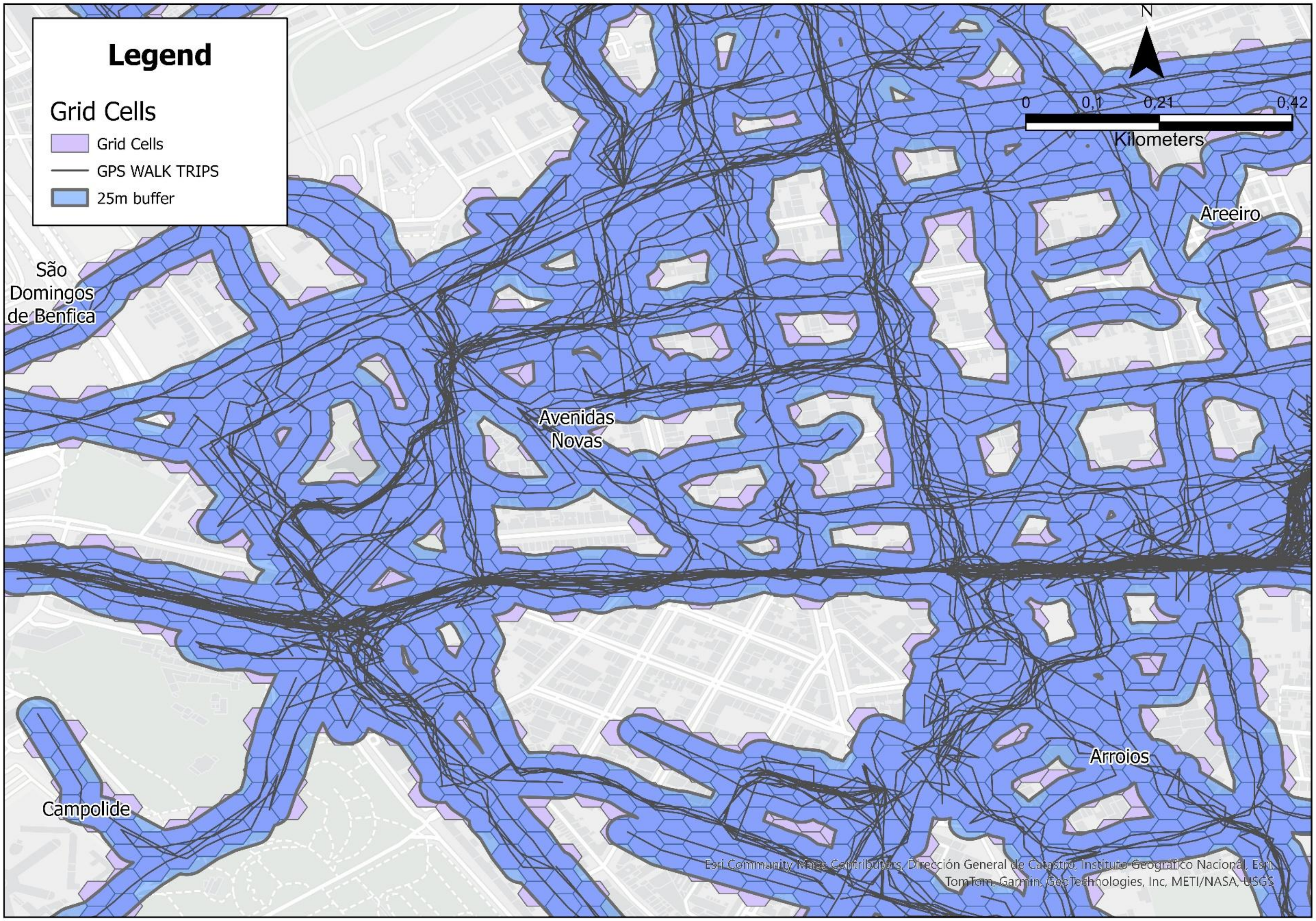

Figure S1. Grid cells and buffered GPS walking paths

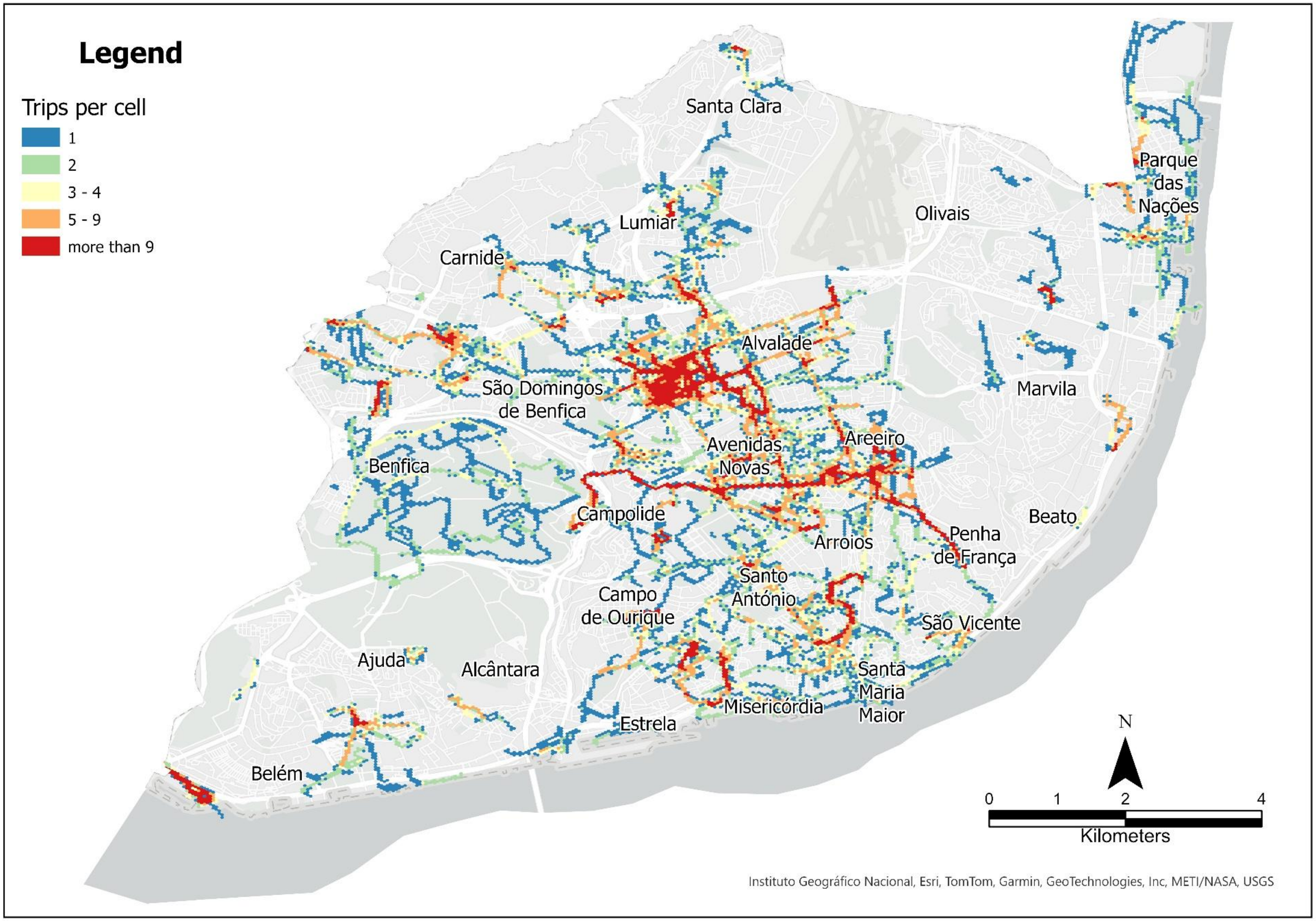

Figure S2. Density of Walking Trips Across the Study Area

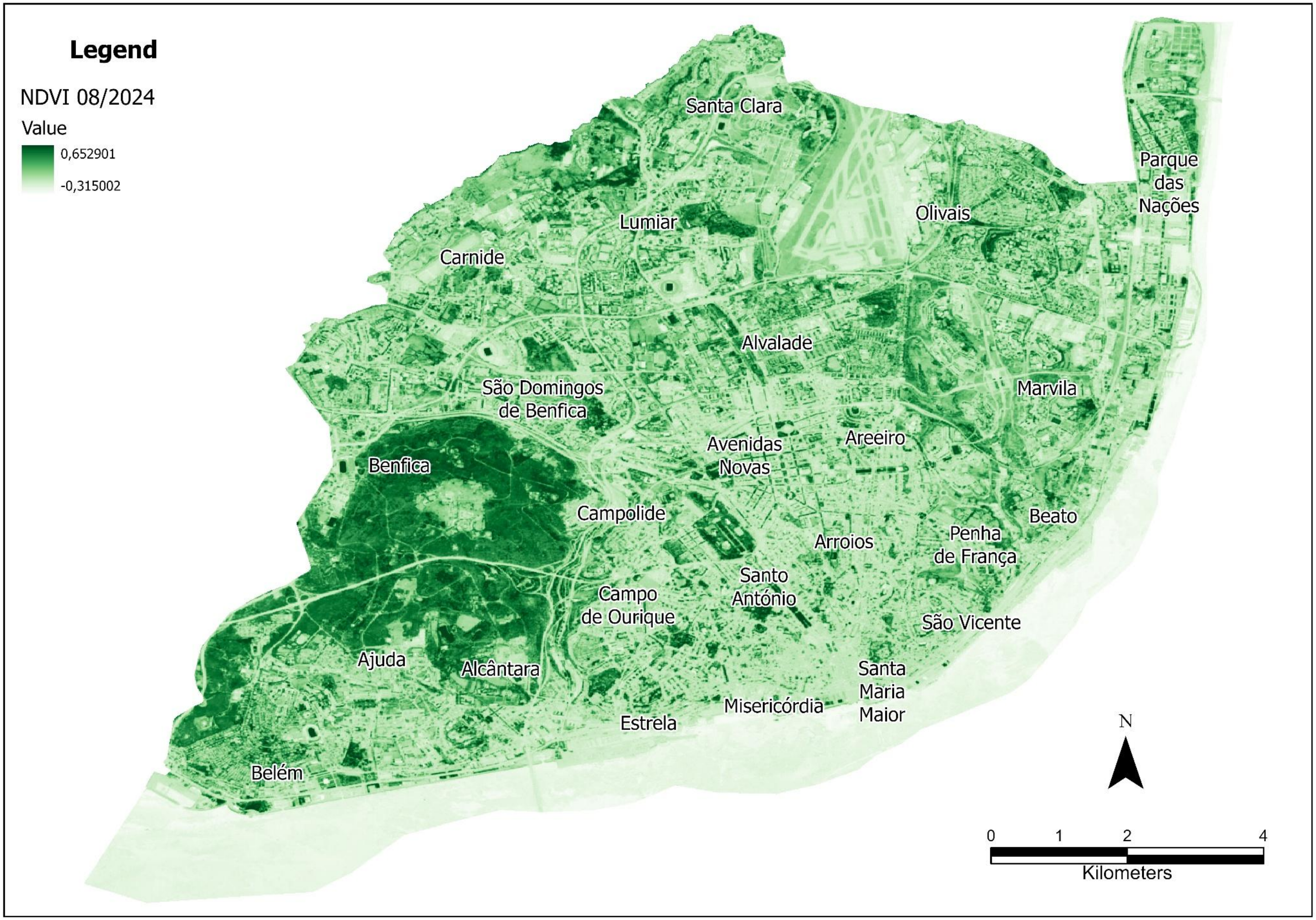

Figure S3. NDVI 08/2024

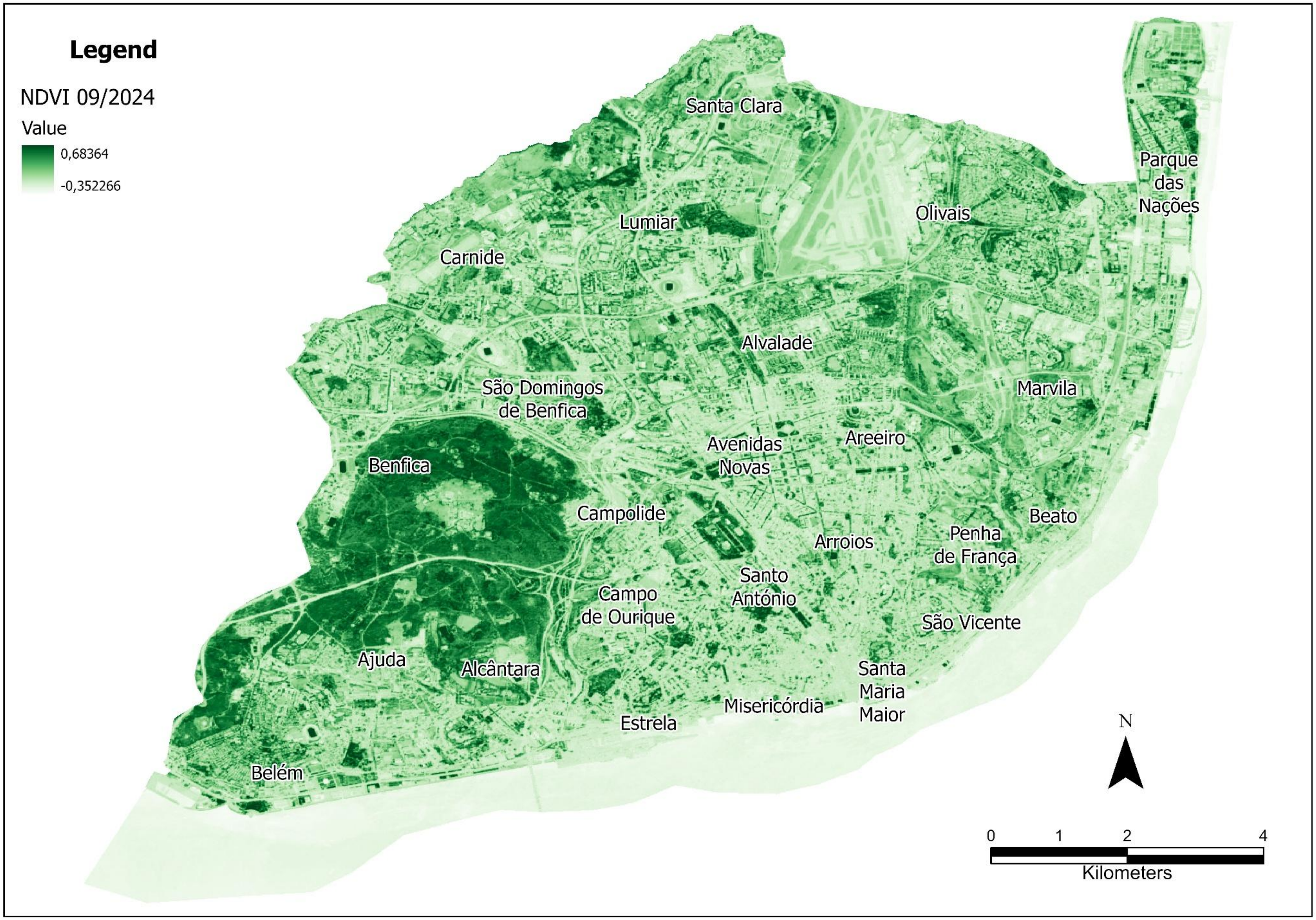

Figure S4. NDVI 09/2024

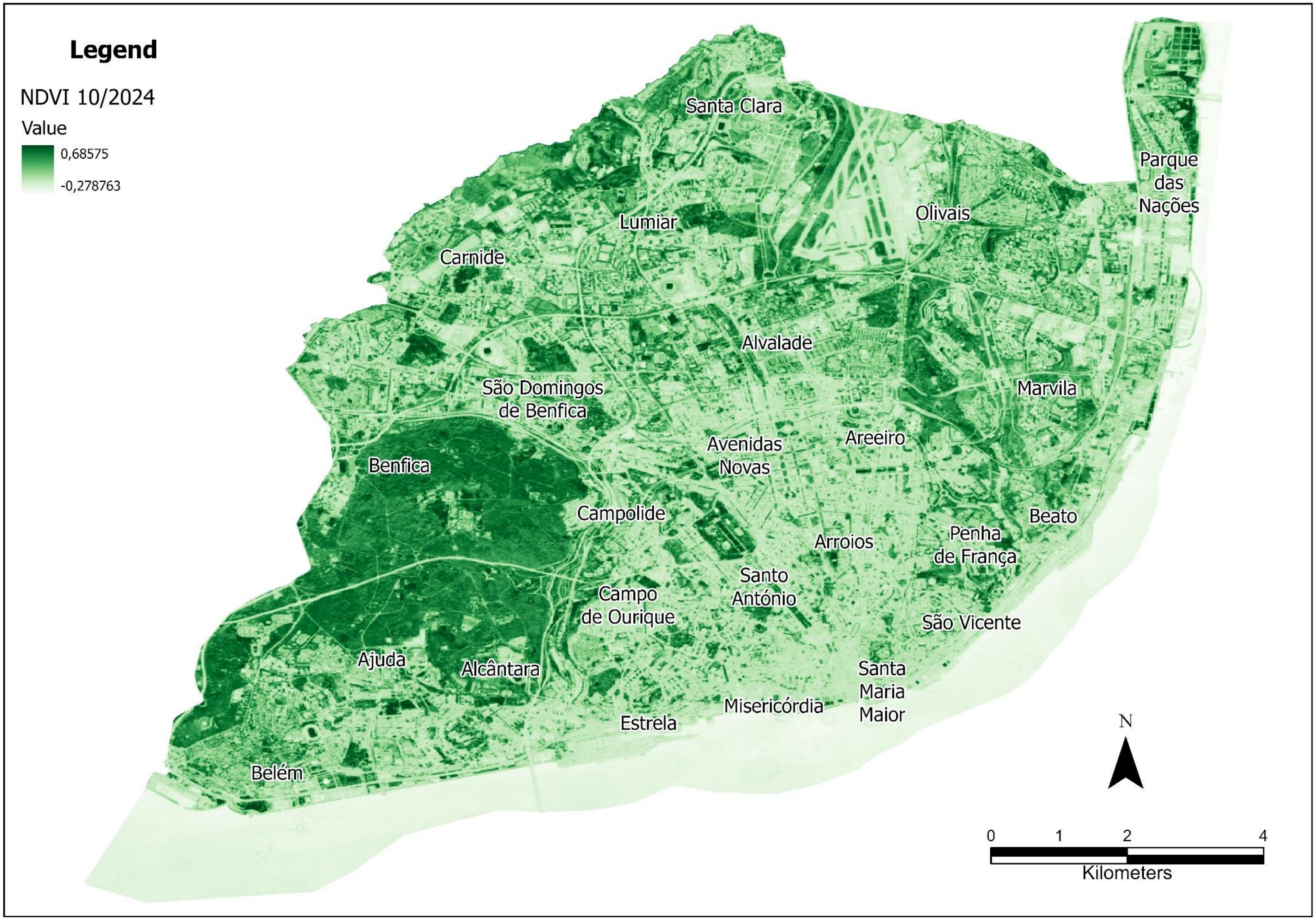

Figure S5. NDVI 10/2024

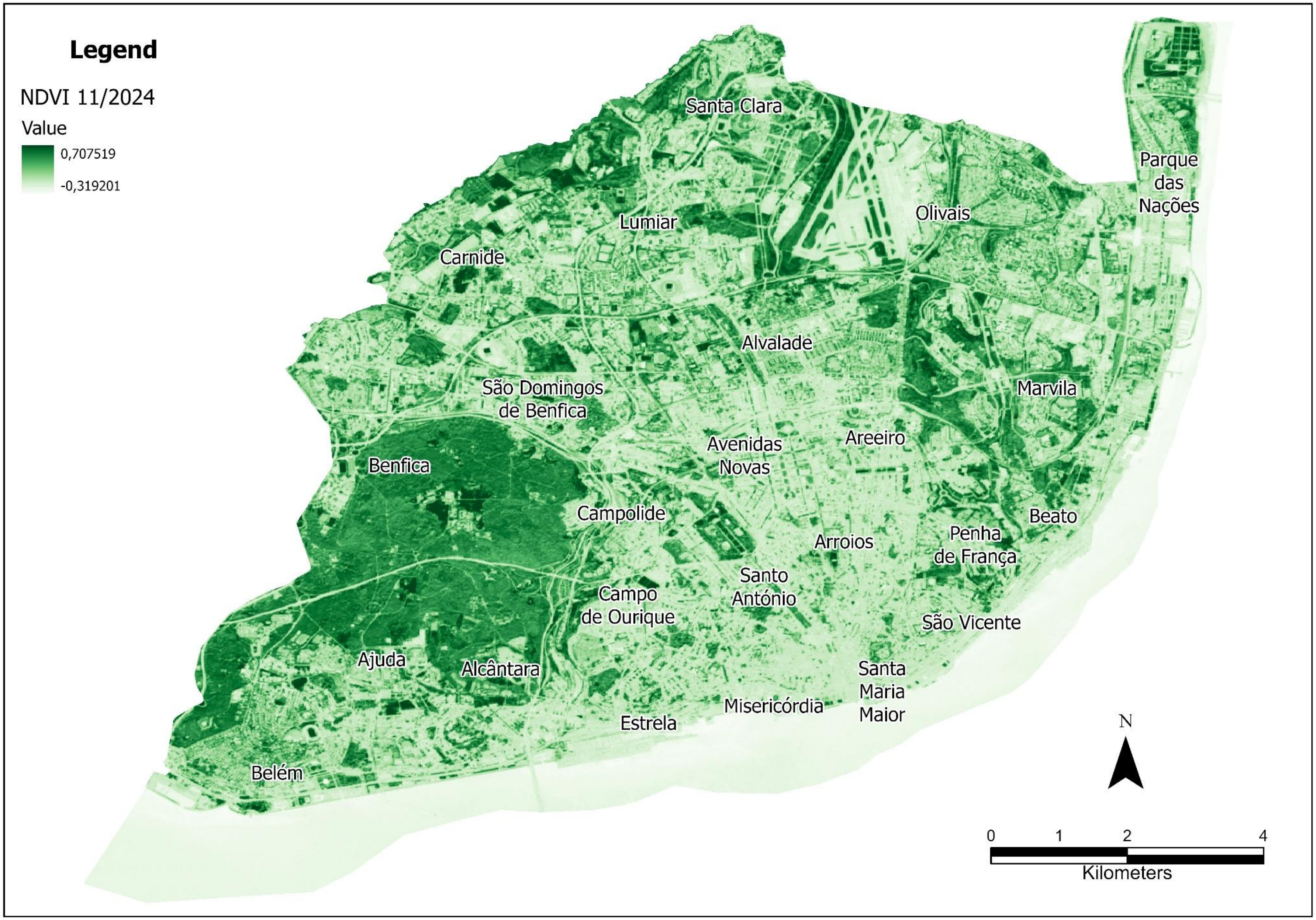

Figure S6. NDVI 11/2024

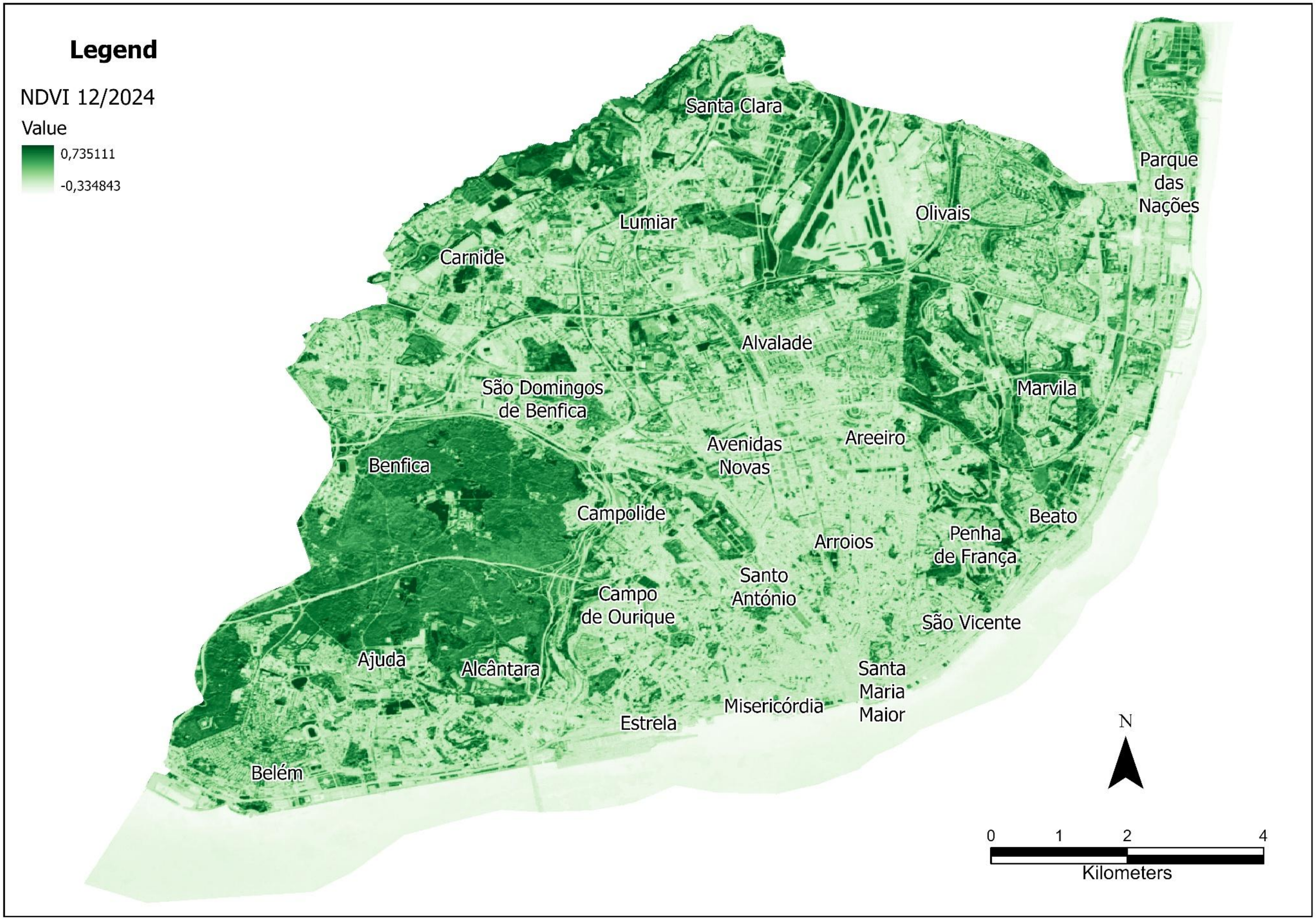

Figure S7. NDVI 12/2024

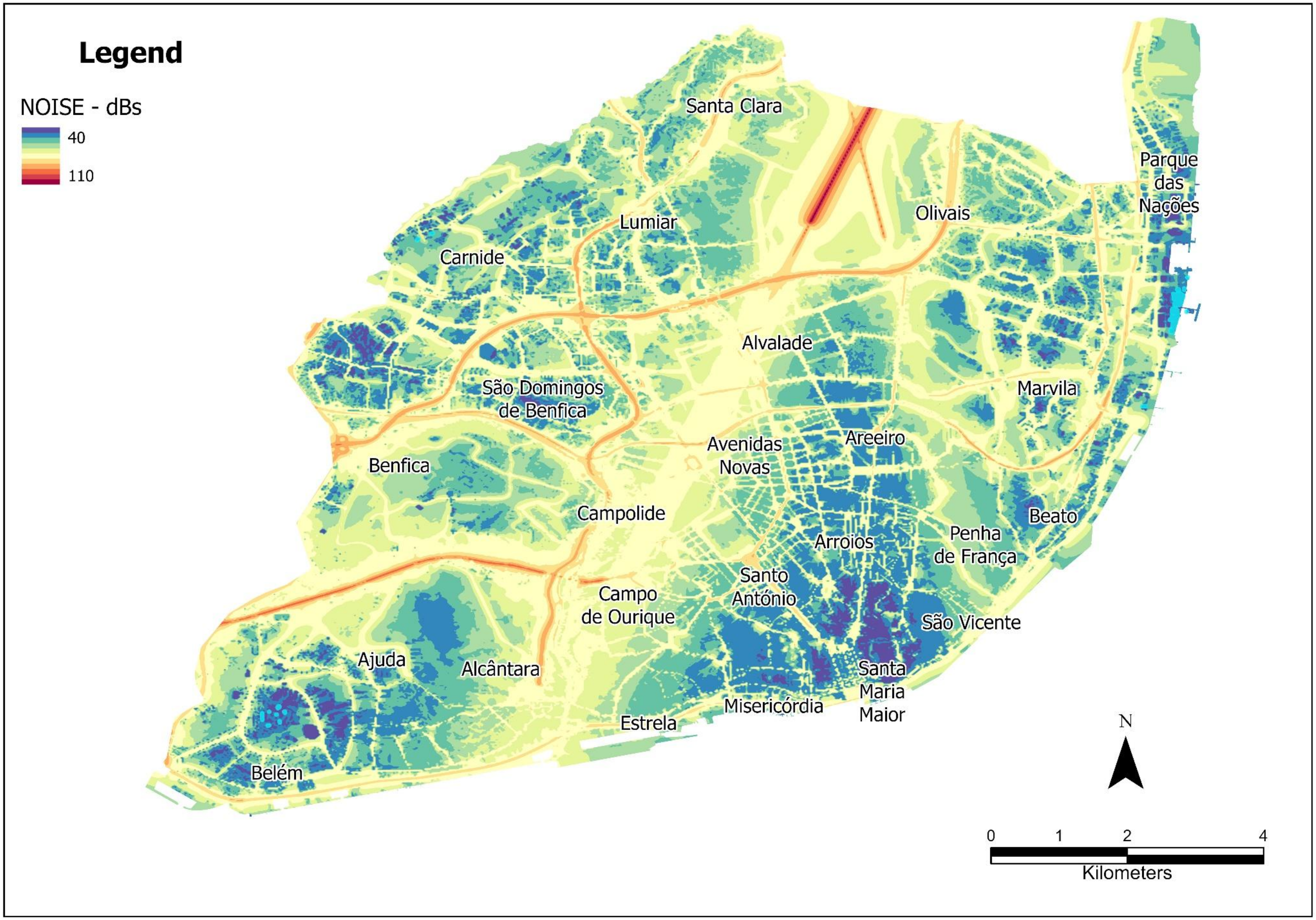

Figure S8. Noise map

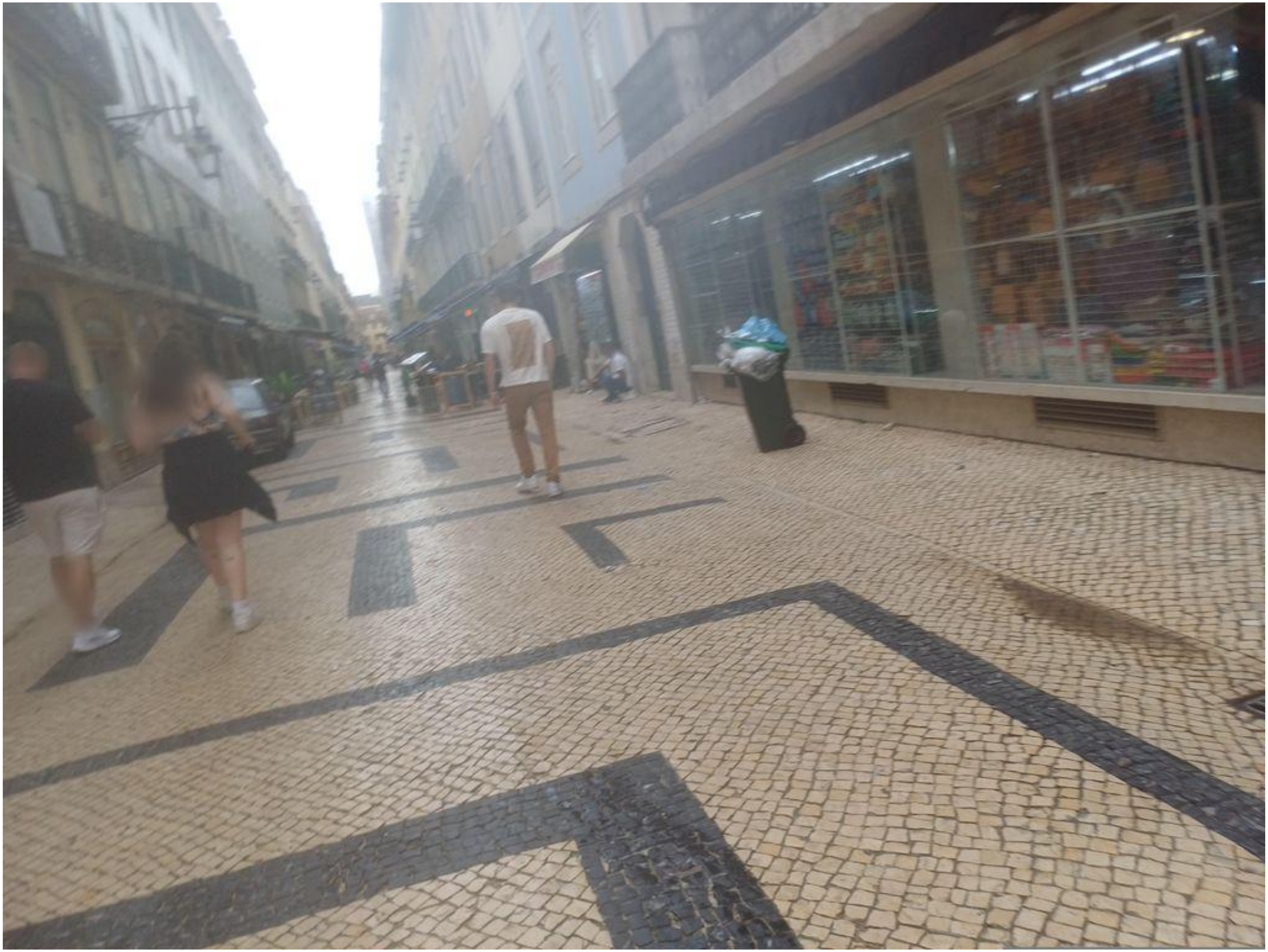

Figure S9. Picture from cluster 0

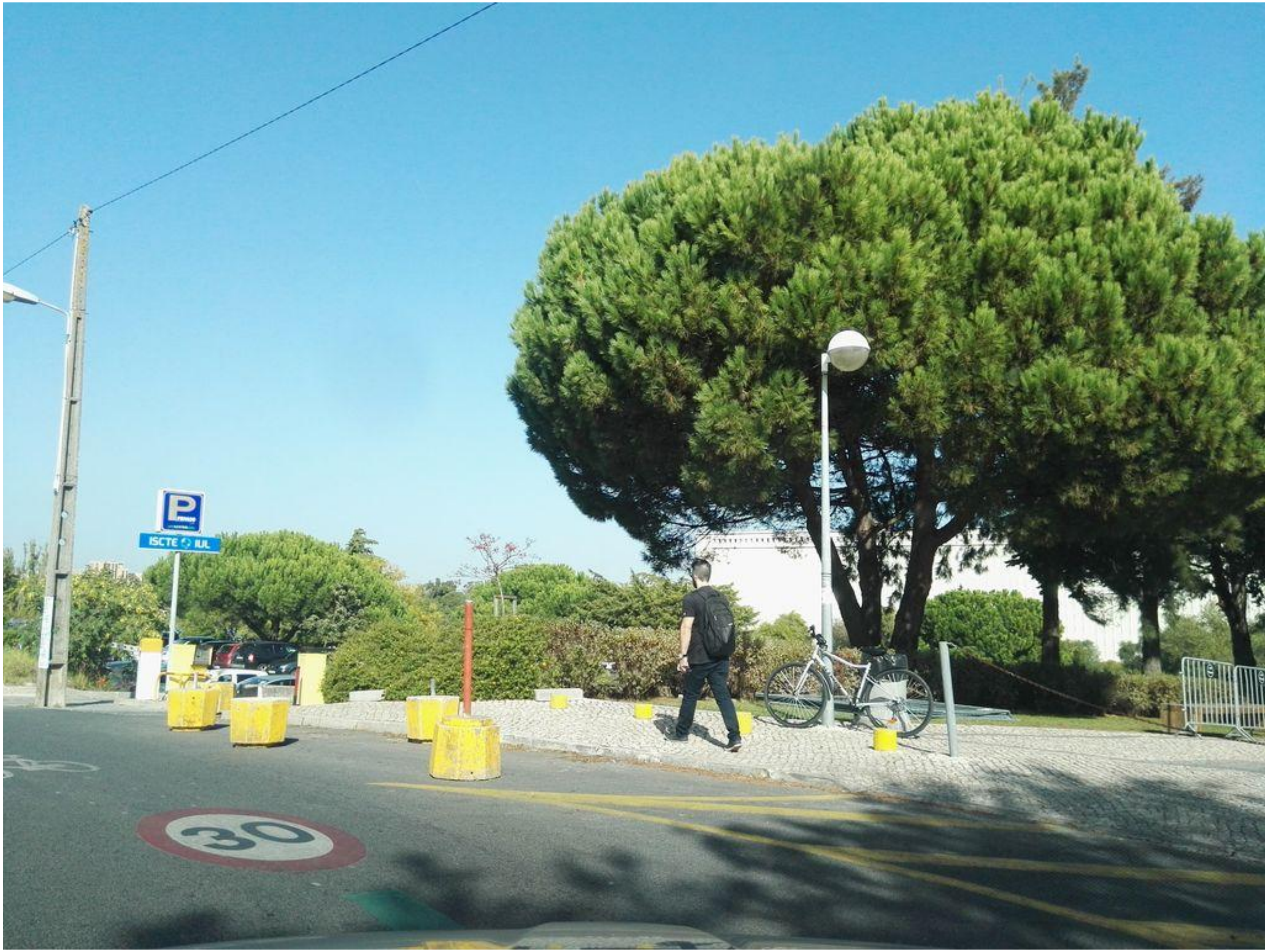

Figure S10. Picture from cluster 1

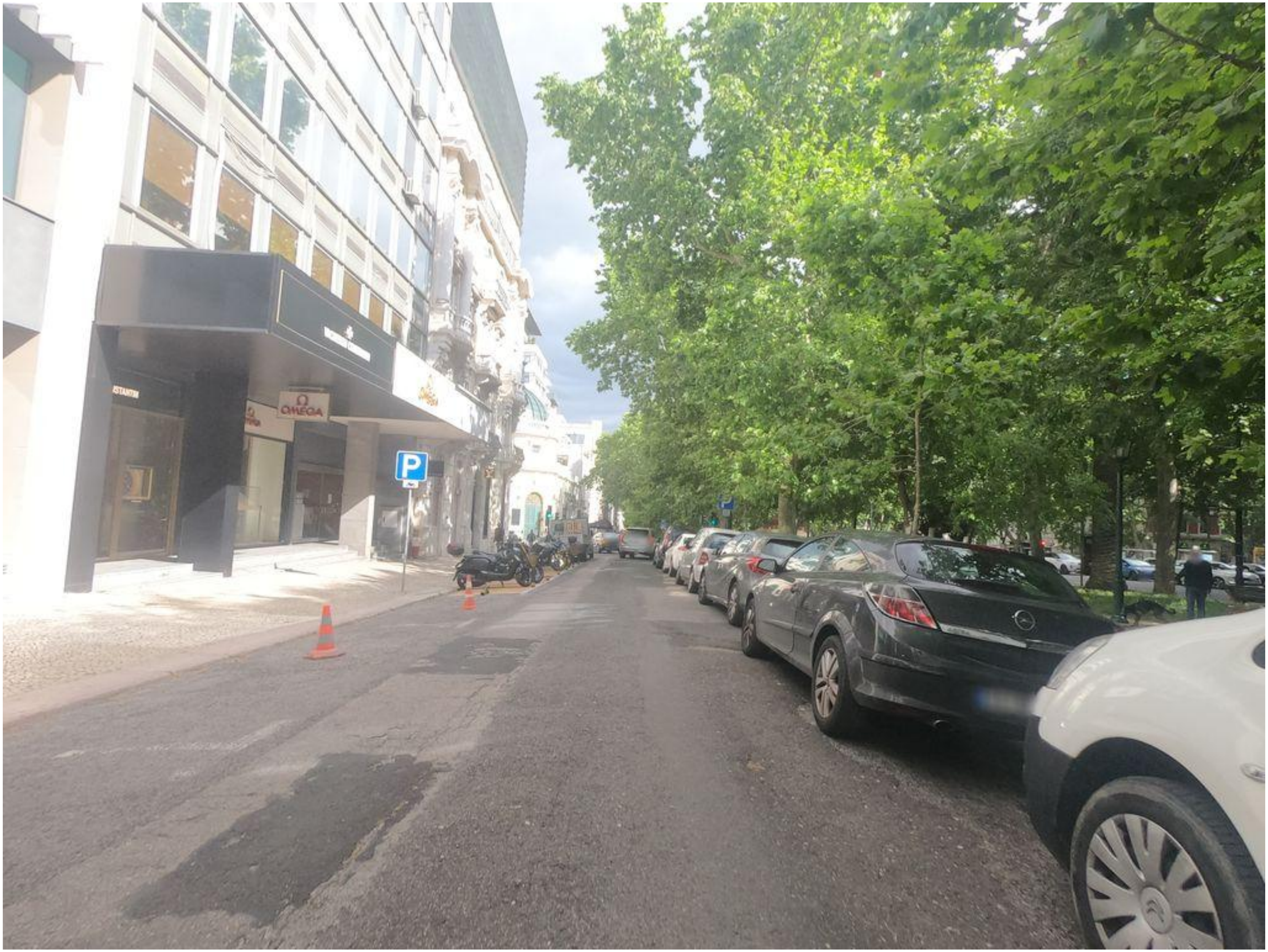

Figure S11. Picture from cluster 2

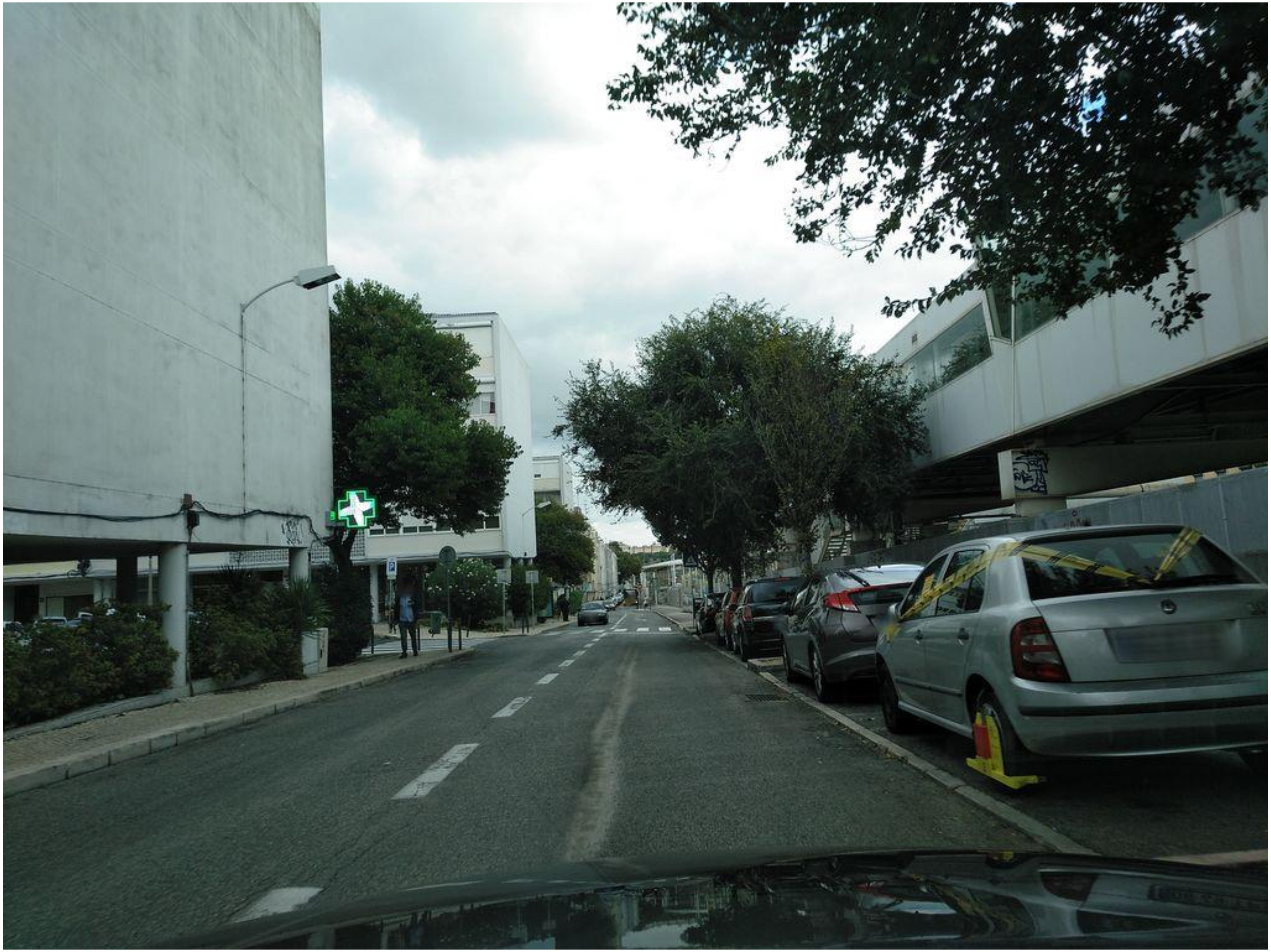

Figure S12. Picture from cluster 3

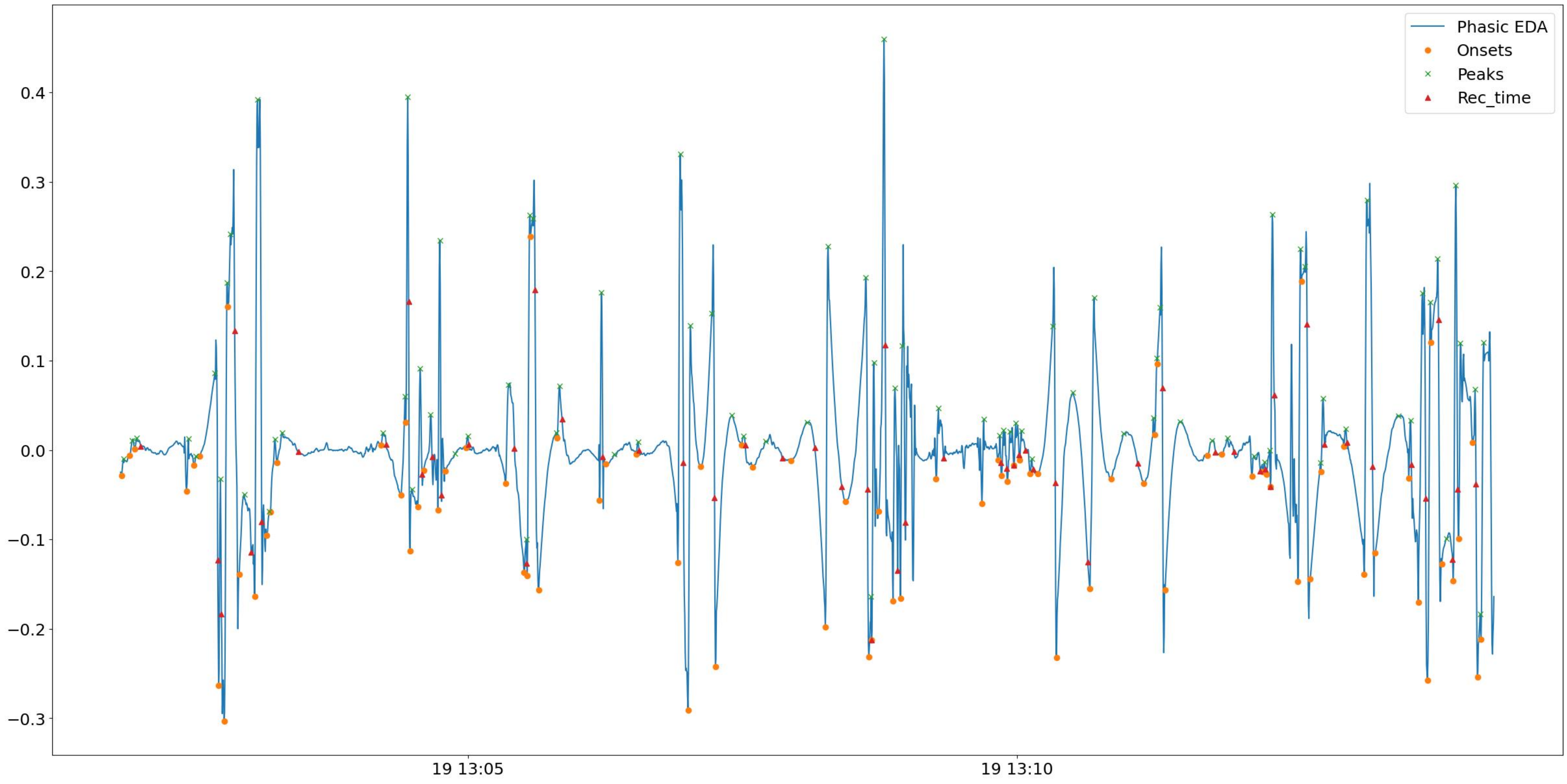

Figure S13a. Phasic EDA signal with identified onsets (orange), peaks (green), and recovery times (red).

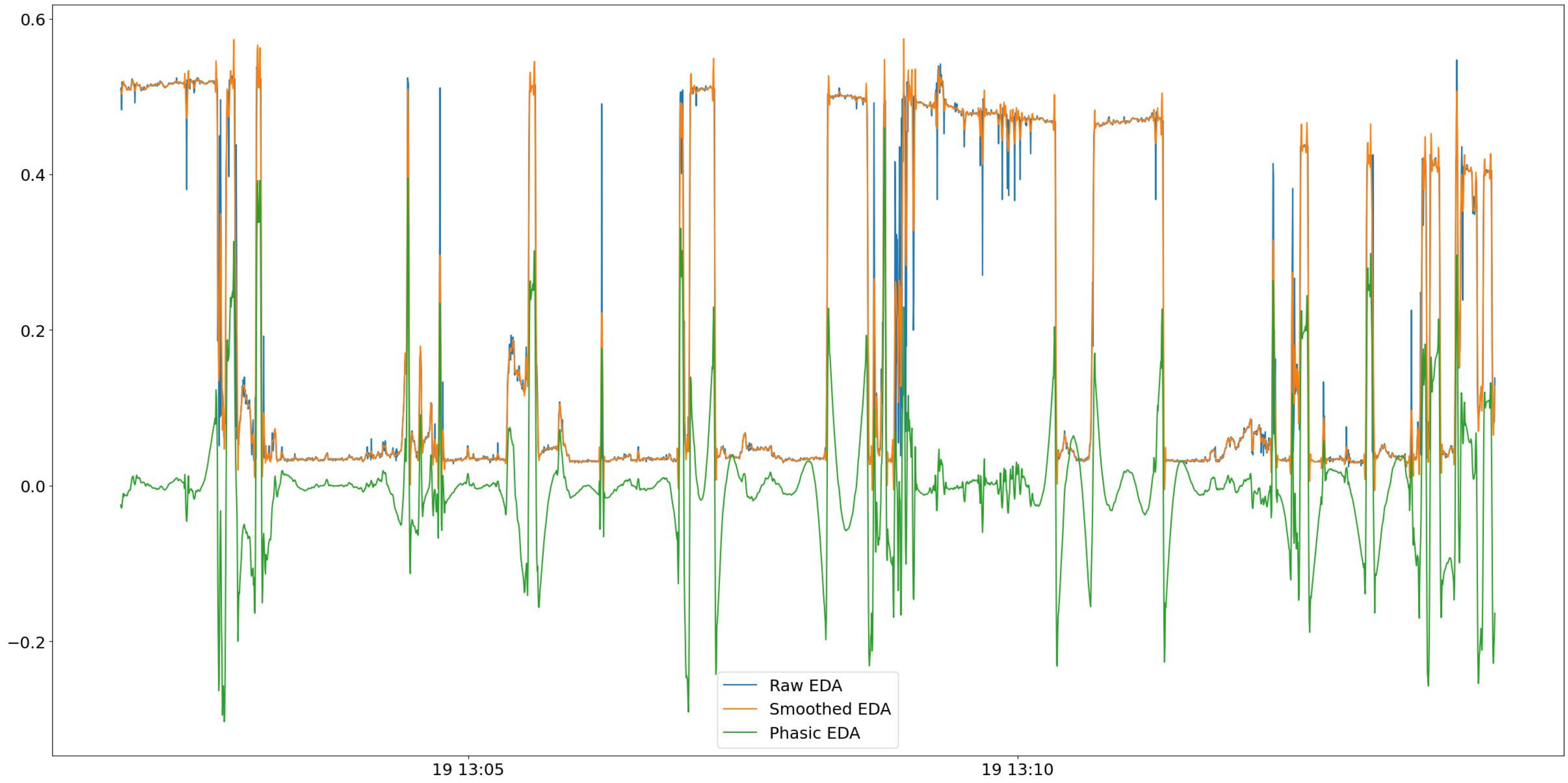

Figure S13b. Phasic EDA signal with identified onsets (orange), peaks (green), and recovery times (red).

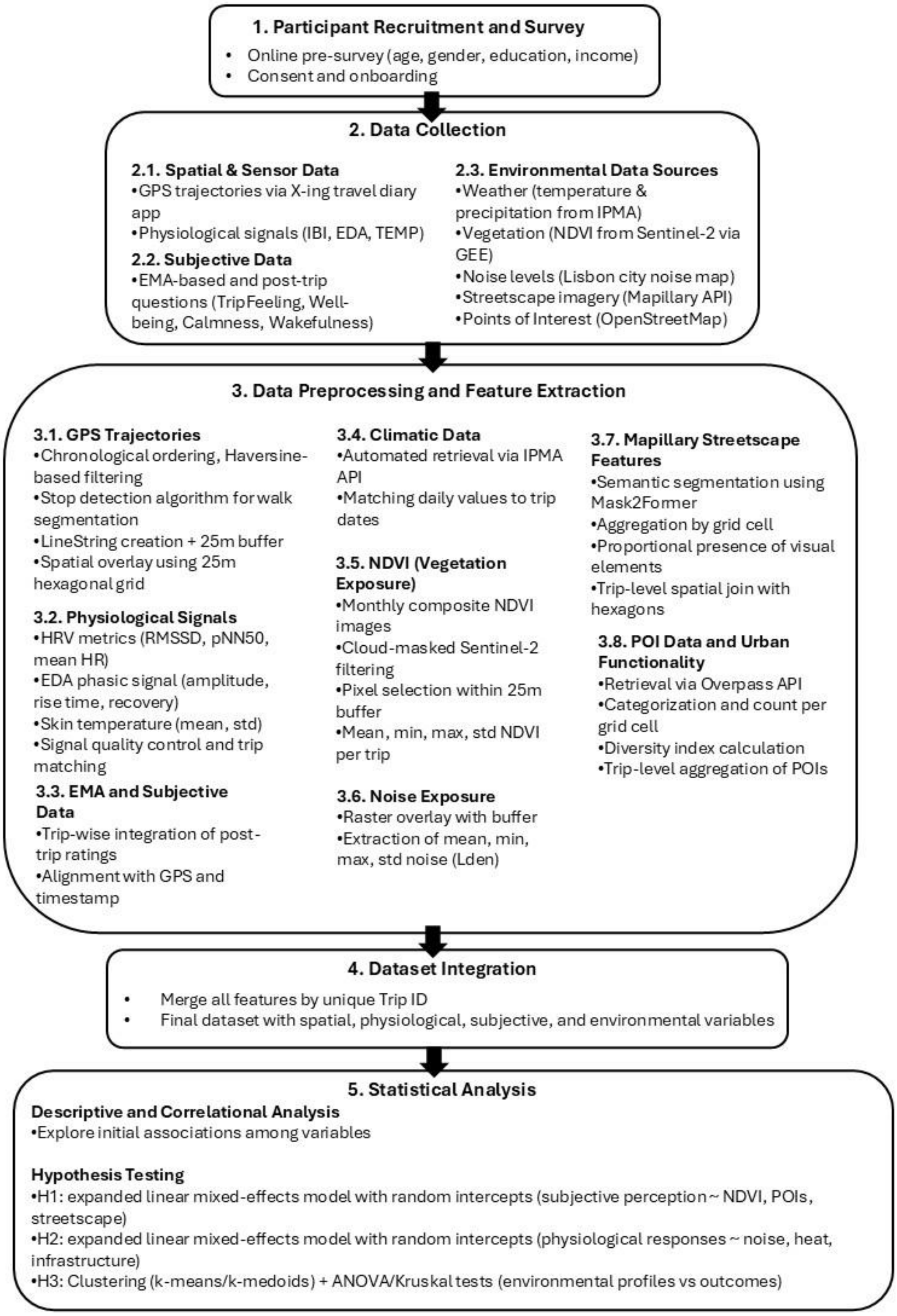

Figure S34. Workflow
