# Supplementary material for: The Influence of Urban Context on Emotions and Bodily Responses During Walking
Source: J Urban Health. 2026 Mar 27;103(2):344–56. doi: 10.1007/s11524-025-01051-1 (PMC13235672; doi:10.1007/s11524-025-01051-1)
Supplement: Supplementary file 2 — (PDF 204 KB) [file 11524_2025_1051_MOESM2_ESM.pdf]

Supplementary Methods

S2.4. Climatic Data Acquisition and Processing

Daily climatic conditions were retrieved from the Portuguese Institute of the Sea and Atmosphere (IPMA), using data from the Lisboa/Gago Coutinho station (code 1200579), which provides daily-resolution meteorological records for the metropolitan area. A Python script (*1.APMA_data.py*, available in the public Zenodo repository) accessed the public API and downloaded maximum, mean, and minimum temperature and total precipitation for the full monitoring period (Aug–Dec 2024).

Weather values were matched to each walking trip using GPS timestamps (day/month/year). Because data come from a single monitoring station, climatic exposure does not vary spatially across Lisbon — all routes recorded on the same day share the same climatic values. These covariates were used to capture effects of day-to-day weather variation in physiological and perceptual outcomes.

S2.5 NDVI Extraction and Processing

Vegetation exposure was quantified using NDVI (Normalized Difference Vegetation Index) derived from Sentinel-2 Surface Reflectance imagery (10 m) processed in Google Earth Engine (GEE). Cloud masking was applied using MSK_CLDPRB <10%, and monthly NDVI composites were generated for August–December 2024 using the clearest pixel approach. Resulting rasters (EPSG:4326) were clipped to the Lisbon extent and exported for analysis.

Each pixel was converted to a centroid point, enabling spatial aggregation. NDVI values were summarized for every walking trip within a 25 m GPS-based buffer, producing mean, min, max, and standard deviation vegetation exposure metrics per route. These trip-level summaries were integrated into the unified analysis dataset and served as continuous environmental predictors for subjective and physiological outcomes. Monthly NDVI maps are illustrated in Supplementary Figures S3–S7 and code for the GEE workflow is fully available in *2.NDVI_LISBON_GEE.js* (Zenodo repository).

S2.6 Noise Pollution Data Processing

Noise exposure was quantified using the official environmental noise map published by *Câmara Municipal de Lisboa* (Lisboa Aberta platform), which provides city-wide Lden indicators expressed in dB(A). Lden represents the 24-hour weighted equivalent sound level, incorporating +5 dB penalties during evening hours and +10 dB during night periods. Noise estimates were generated using CadnaA modeling software and include emissions from major traffic, rail, aviation, and industrial sources.

The polygon-based noise layer was rasterized to a 4 m resolution, and centroid values were intersected with buffered GPS tracks (25 m) and the hexagonal grid used for contextual exposure mapping. For each route, we computed the mean, min, max, and SD Lden, enabling fine-scale variation even between trips in adjacent areas.

The spatial distribution of modeled noise across Lisbon is shown in Supplementary Figure S8, and the full processing workflow is documented in the Supplementary code archive.

S2.7 Streetscape Feature Extraction from Mapillary Imagery

Streetscape characteristics were extracted from Mapillary, a georeferenced street-level imagery platform. A custom Python script automated image retrieval from the Mapillary API, collecting the 10 nearest images per 25-m hexagonal grid cell, resulting in 462,964 images (~100.8 GB) indexed by spatial ID.

Semantic segmentation was performed using the Mask2Former architecture pretrained on the Mapillary Vistas dataset, which provides state-of-the-art accuracy for pixel-level classification of urban scenes (high mIoU, precision, and recall). The procedure follows and expands previous work applying segmented Mapillary imagery to environmental exposure estimation in European cities.

For each image, pixels were assigned to semantic categories (vegetation, sky, roads, sidewalks, poles, vehicles, etc.). Feature values were aggregated per grid-cell and summarized for each walking route by intersecting cells whose centroids overlapped the 25-m trajectory buffer. Mean pixel-proportion per class was calculated as:

$$F_c = \frac{1}{N}\sum_{i=1}^{N}\left(\frac{P_{c,i}}{T_i}\right)$$

where Fc is the proportion of class c along the route, Pc,i is the pixel count for class c in cell i, Ti is the total pixel count, and N is the number of intersecting cells. These continuous proportional measures were incorporated as visual exposure predictors in the statistical models.

The full pipeline for image retrieval and semantic classification is documented in the scripts "3.Mapillary_collect.py" and "4.Mapillary_segmetation.py", which are publicly available in Zenodo repository.

S2.8. Points of Interest (POI) from OpenStreetMap

Functional diversity along walking routes was evaluated using Points of Interest (POIs) obtained from OpenStreetMap. A custom Python script retrieved nodes, ways, and

relations from the Overpass API for each cell of a 50-m hexagonal grid, selecting all elements located within 25 m of each centroid.

POIs were grouped into thematic categories (e.g., retail & services, food & drink, tourism, buildings & facilities, greenery, public services) based on OSM key–value tags using a classification structure consistent with previous work. For each grid cell, category-specific POI counts were computed, and terrain slope was estimated where elevation metadata was present. POIs were then associated with walking trips by selecting grid cells whose centroids intersected each 25-m route buffer. Final aggregated exposure per route was calculated as:

$$F_c = \sum_{i=1}^{N} P_{c,i}$$

where Fc is the total number of POIs in category c along the route, Pc,i is the number of POIs in cell i, and N is the number of intersecting grid cells. These aggregated indicators — including POI totals, functional diversity index, and estimated slope — were merged into the master dataset and used as explanatory predictors in subsequent statistical models. Full implementation is available at 5.Osm_data.py (Zenodo repository).
