# Supplementary material for: The Influence of Urban Context on Emotions and Bodily Responses During Walking
Source: J Urban Health. 2026 Mar 27;103(2):344–56. doi: 10.1007/s11524-025-01051-1 (PMC13235672; doi:10.1007/s11524-025-01051-1)
Supplement: Supplementary file 3 — (PDF 458 KB) [file 11524_2025_1051_MOESM3_ESM.pdf]

	Feelings			
	Trip Feeling Environment	Agited - Calm	Tired - Awake	Unwell - Well
**Sociodemographic Data**				
AGE	0.33	0.11	0.15	0.12
**Physiological Data**				
STD rise time npoints ratio frequency			0.12	
STD RMSSD	0.12			-0.10
MAX LF HF ratio	0.11			
MAX pNN50	0.11			
MAX rise time npoints ratio frequency			0.13	
MAX RMSSD				-0.10
MAX Values mean frequency			-0.10	
MEAN rise time npoints ratio frequency			0.12	
MEAN Values frequency			-0.10	
MIN bpm frequency			0.12	0.12
**Climatic Data**				
MAX Temperature	0.13			
MEAN Temperature	0.12			
MIN Temperature	0.11			
**NDVI**				
MEAN NDVI		0.14	0.13	
MIN NDVI	-0.11			
**Noise**				
MIN NOISE		-0.10		
**Streetscape Feature (Mapillary)**				
Traffic Light	-0.12			
Utility Pole		-0.10	-0.11	
Building				0.10
Curb	-0.15	0.16		0.11
Fence				0.12
Manhole	-0.12			
Person	0.10			
Pole	-0.10			
Sky		-0.11		
**Points of Interest (OSM)**				
Slope	0.16			
arts entertainment events	0.12			
beauty personal health			-0.11	-0.10
food drink		-0.11	-0.10	
greenery natural	0.12			
miscellaneous services		-0.17	-0.16	-0.12
amenity	0.11			
buiding		-0.13		-0.11
leisure	0.21	0.30	0.24	0.25
shop	-0.11	-0.14	-0.21	
tourism	0.25			
transportation		-0.19	-0.13	-0.11
public services			-0.11	-0.12
roads transportation				-0.10
water body	0.16			-0.14

Table S1. Bivariate Pearson Correlations Between Environmental Indicators and Self-Reported Feelings During Pedestrian Routes

Note: Values represent Pearson correlation coefficients (r) calculated at the trip level (n = 2,207 walking trips). "Trip Feeling Environment" reflects participants' self-reported rating (0–5 scale) of how the surrounding environment influenced their mood after each trip. "Unwell–Well," "Agitated–Calm," and "Tired–Awake" are self-reported affective scales ranging from 0 (negative pole) to 5 (positive pole). Environmental indicators (e.g., NDVI, noise, sky visibility, points of interest, and street features) were aggregated for each trip and standardized (mean = 0, SD = 1) prior to analysis. Correlations were computed using pairwise complete observations. Positive values indicate that higher environmental scores are associated with more positive self-reported feelings.

Predictor	Trip Feeling Environment (β)	Trip Feeling Environment (p)	Unwell-Well (β)	Unwell-Well (p)	Agitated-Calm (β)	Agitated-Calm (p)	Tired-Awake (β)	Tired-Awake (p)
**NDVI mean**	-0.04	0.458	0.029	0.581	0.126	0.031	0.129	0.047
**NDVI max**	-0.023	0.596	-0.051	0.228	-0.094	0.048	-0.066	0.216
**POI leisure**	0.015	0.75	0.03	0.523	0.034	0.517	0.01	0.866
**POI tourism**	0.047	0.209	0.049	0.174	0.08	0.052	0.127	0.006
**Vegetation**	-0.017	0.709	0.019	0.658	0.033	0.501	0.038	0.487
**Sky**	0.004	0.922	-0.064	0.089	-0.115	0.006	-0.019	0.693
**Curb**	-0.001	0.971	0.078	0.046	0.092	0.036	0.099	0.042
**Arts entertainment events**	0.035	0.262	-0.003	0.912	-0.009	0.796	0.019	0.614
**Greenery natural**	0.049	0.194	-0.008	0.832	-0.035	0.397	-0.069	0.134
**Water body**	0.023	0.522	-0.073	0.037	-0.055	0.161	-0.07	0.112
**Slope**	0.044	0.186	0.032	0.311	0.044	0.224	0.011	0.789

Table S2. Mean Differences in Self-Reported Feelings Associated with a Unit Increase in Environmental Indicators (Linear Mixed-Effects Models, Trip Level)

Note: Results represent standardized regression coefficients (β) and corresponding p-values from linear mixed-effects models with random intercepts for participants (n = 90 individuals; 2,207 walking trips). Outcome variables correspond to self-reported feelings rated on 0–5 semantic differential scales: “Trip Feeling Environment,” “Unwell–Well,” “Agitated–Calm,” and “Tired–Awake.” Predictors include environmental and infrastructural indicators aggregated at the trip level within a 25 m buffer around the GPS trajectory. Continuous variables (e.g., NDVI, noise, temperature) were averaged, and categorical indicators (e.g., presence of POIs, street features) were expressed as relative densities (number of occurrences per trip area). All predictors were standardized (mean = 0, SD = 1), such that coefficients represent mean differences in Y associated with a one standard deviation increase in X. Models were not mutually adjusted; each predictor was tested in a separate model controlling for participant-level clustering via random intercepts.

	Electrodermal Activity									Interbeat Interval																Skin Temperature		
	STD Amplitude	STD Rise time Npoints	MAX Amplitude	MAX Rise time	MAX Rise Time Npoints	MEAN Amplitude	Mean Rise time npoints	MIN Amplitude	MIN Rise Time	STD bpm	STD LF_HF ratio	STD pNN50	STD RMSSD	MAX bpm	MAX LF_HF ratio	MAX pNN50	MAX RMSSD	MEAN bpm	MEAN LF_HF ratio	MEAN pNN50	MEAN RMSSD	MIN bpm	MIN LF_HF ratio	MIN pNN50	MIN RMSSD	STD Values	MAX Values	MIN Values
**Trip data**																												
Trip Distance	0.15		0.36	0.28	0.22	0.15				0.13	0.12	0.15	0.17	0.2	0.18	0.14	0.22					-0.16		-0.15	-0.15	0.49		-0.27
Trip minutes	0.15	0.14	0.36	0.35	0.3	0.13	0.13			0.14	0.16	0.21	0.23	0.21	0.24	0.19	0.29					-0.22		-0.19	-0.19	0.55		-0.3
Speed		-0.1			-0.15				0.12			-0.13	-0.14				-0.1					0.14		0.1	0.12	-0.14		
**Sociodemographic Data**																												
Study level																					-0.12				-0.14			
**Subjective Feelings**																												
Trip Feelings Environment													0.12		0.11	0.11												
Unwell - Well													-0.1				-0.1					0.12						
Tired - Awake		0.12			0.13		0.12															0.12					-0.1	
**Climatic Data**																												
MAX Temperature	0.17		0.17			0.22				-0.12										-0.13	-0.14				-0.12		0.16	0.16
MEAN Temperature	0.16		0.16			0.23														-0.12	-0.13				-0.13		0.19	0.17
MIN Temperature	0.13		0.12			0.19															-0.1				-0.12		0.18	0.15
**NDVI**																												
MAX NDVI				0.13						0.12		0.1	0.11	0.16		0.15	0.13									0.23		-0.2
MEAN NDVI																												-0.1
MIN NDVI																										-0.13		
STD NDVI																												-0.1
**Noise**																												
MAX Noise				0.13	0.11							0.14	0.15		0.11	0.13	0.17					-0.14		-0.11		0.23		-0.11
Mean Noise																										0.11		
Min Noise															-0.12			0.15	-0.1			0.12						
STD Noise																		-0.15				-0.13						
**Streetscape Feature (Mapillary)**																												
Car														0.2				0.17										
Curb																						-0.1					0.1	
Manhole																											0.1	0.12
Slope		0.1												0.11			0.16											
Utility_Pole																											0.14	0.13
Vegetation							-0.12																					
**Points of Interest (OSM)**																												
Arts entertainment events											0.12				0.13													
Beauty personal health		-0.2			-0.19		-0.19			-0.17			-0.1			-0.11	-0.15	0.1		-0.12	-0.14	0.16					0.16	0.15
Buildings facilities					0.17		0.17	-0.1						0.12												0.29		-0.23
Food drink		-0.1																							-0.1			
Greenery natural			0.13	0.11							0.11			0.15	0.15				0.13	-0.1				-0.14	-0.12	0.2		-0.14
Miscellaneous services													-0.1	0.12			-0.1	0.14								0.1		
Amenity										0.12				0.12	0.11							-0.2						
Buiding																							-0.11					-0.11
Historic			0.12																	-0.1		-0.12				0.19		-0.15
Shop		-0.15			-0.13		-0.13																				0.21	0.19
Tourism		-0.11	0.17	0.12		0.13					0.13				0.12			0.11	0.12	-0.11	-0.13				-0.1	0.12		-0.14
Transportation														0.13				0.14								0.11		-0.1
Public services				0.13																-0.14	-0.13			-0.11	-0.13			
Roads transportation								-0.12			0.15				0.18				0.15			-0.15		-0.13	-0.12	0.33		-0.18
Tourism leisure				0.11		0.12																				0.13		-0.12
Water body														0.12								-0.11				0.13		-0.15

Table S3.Bivariate Pearson Correlations Between Environmental Variables, Walking Behavior, and Physiological Responses

Note: Values represent Pearson correlation coefficients (r) between environmental characteristics, walking behavior, and physiological indicators, computed at the trip level (n = 2,207 walking trips). Environmental variables—including vegetation indices (NDVI), climatic conditions (temperature), noise exposure, streetscape features (from Mapillary imagery), and points of interest (from OpenStreetMap)—were aggregated within a 25 m buffer around each GPS trajectory. Continuous variables (e.g., NDVI, noise, temperature) were averaged, while categorical or count-based features (e.g., points of interest, streetscape elements) were converted to densities. All environmental predictors were normalized to a 0–1 range prior to analysis.

Physiological indicators (columns) represent trip-level averages of electrodermal activity (EDA), interbeat interval–derived heart rate variability (HRV) metrics, and skin temperature features collected by the Empatica E4 wristband. Walking behavior variables (rows) include total distance, duration, and mean speed per trip. Positive correlations indicate that higher environmental or behavioral values are associated with higher physiological activation or more favorable environmental exposure, while negative correlations denote inverse relationships.

System	Block	Dependent variable	Predictor	Coefficient (β)	p-value
EDA	Infrastructure	MAX_amplitude	Group Var	0.2067	0.0003
EDA	Infrastructure	MAX_rise_time	Group Var	0.1981	0.0025
EDA	Infrastructure	MAX_rise_time_npoints	Group Var	0.3837	0.0001
EDA	Infrastructure	MAX_rise_time_npoints	Manhole	-0.002	0.038
EDA	Infrastructure	MEAN_amplitude	Group Var	0.1857	0.0002
EDA	Infrastructure	MEAN_rise_time_npoints	Building	0.0009	0.0458
EDA	Infrastructure	MEAN_rise_time_npoints	Manhole	-0.0009	0.0447
EDA	Infrastructure	MIN_amplitude	Group Var	0.0956	0.0292
EDA	Infrastructure	STD_amplitude	Group Var	0.2484	0.0004
EDA	Infrastructure	STD_rise_time_npoints	Group Var	0.2616	0.0004
EDA	Infrastructure	STD_rise_time_npoints	Pole	0.0006	0.0295
EDA	Infrastructure	STD_rise_time_npoints	Traffic_Light	-0.0005	0.0493
EDA	NOISE	MAX_amplitude	Group Var	0.192	0.0003
EDA	NOISE	MAX_rise_time	Group Var	0.1456	0.0011
EDA	NOISE	MAX_rise_time	NOISE_max	0.2538	0.0026
EDA	NOISE	MAX_rise_time_npoints	Group Var	0.3708	0.0001
EDA	NOISE	MEAN_amplitude	Group Var	0.1817	0.0002
EDA	NOISE	MEAN_rise_time_npoints	NOISE_std	0.001	0.0231
EDA	NOISE	MIN_amplitude	Group Var	0.1083	0.0275
EDA	NOISE	STD_amplitude	Group Var	0.2381	0.0005
EDA	NOISE	STD_rise_time_npoints	Group Var	0.2471	0.0005
EDA	POI	MAX_rise_time	Group Var	0.1501	0.0005
EDA	POI	MAX_rise_time_npoints	Group Var	0.3766	0.0001
EDA	POI	MAX_rise_time_npoints	public_services	0.0031	0.0141
EDA	POI	MEAN_amplitude	Group Var	0.1734	0.0002
EDA	POI	MEAN_rise_time_npoints	public_services	0.0012	0.0443
EDA	POI	MIN_amplitude	Group Var	0.0959	0.0284
EDA	POI	STD_amplitude	Group Var	0.2251	0.0004
EDA	POI	STD_rise_time_npoints	Group Var	0.2427	0.0005
EDA	Temperature	MAX_amplitude	Group Var	0.1806	0.0003
EDA	Temperature	MAX_rise_time	Group Var	0.2749	0.0173
EDA	Temperature	MAX_rise_time_npoints	Group Var	0.3686	0.0001
EDA	Temperature	MEAN_amplitude	Group Var	0.1638	0.0002
EDA	Temperature	MIN_amplitude	Group Var	0.0911	0.0346
EDA	Temperature	STD_amplitude	Group Var	0.2127	0.0005
EDA	Temperature	STD_rise_time_npoints	Group Var	0.2442	0.0005
EDA	Temperature	STD_rise_time_npoints	Temp_max	-0.0029	0.0021
EDA	Temperature	STD_rise_time_npoints	Temp_mean	0.0027	0.003
EDA	Visuals-Mapillary	MAX_amplitude	Group Var	0.2105	0.0003
EDA	Visuals-Mapillary	MAX_rise_time	Group Var	0.1509	0.001
EDA	Visuals-Mapillary	MEAN_amplitude	Fence	0.0143	0.0424
EDA	Visuals-Mapillary	MEAN_amplitude	Group Var	0.1873	0.0002
EDA	Visuals-Mapillary	MIN_amplitude	Group Var	0.0972	0.0277
EDA	Visuals-Mapillary	STD_amplitude	Group Var	0.2425	0.0004
EDA	Visuals-Mapillary	STD_rise_time_npoints	Fence	-0.0006	0.0323
EDA	Visuals-Mapillary	STD_rise_time_npoints	Group Var	0.2446	0.0004
IBI/HRV	Infrastructure	MAX_bpm	Group Var	0.2456	0.0001
IBI/HRV	Infrastructure	MAX_LF_HF_ratio	Curb	0.0968	0.0168
IBI/HRV	Infrastructure	MAX_LF_HF_ratio	Group Var	0.052	0.0349
IBI/HRV	Infrastructure	MAX_pNN50	Group Var	0.1044	0.0033
IBI/HRV	Infrastructure	MAX_RMSSD	Group Var	0.1014	0.0036
IBI/HRV	Infrastructure	MEAN_bpm	Group Var	0.3088	0.0002
IBI/HRV	Infrastructure	MEAN_LF_HF_ratio	Building	-0.0373	0.0074
IBI/HRV	Infrastructure	MEAN_LF_HF_ratio	Curb	0.0579	0.0013
IBI/HRV	Infrastructure	MEAN_LF_HF_ratio	Group Var	0.0916	0.019
IBI/HRV	Infrastructure	MEAN_LF_HF_ratio	Pole	-0.0299	0.0277
IBI/HRV	Infrastructure	MEAN_pNN50	Traffic_Light	-0.0192	0.047
IBI/HRV	Infrastructure	MEAN_RMSSD	Group Var	0.2013	0.0468
IBI/HRV	Infrastructure	MEAN_RMSSD	Manhole	-2.7588	0.0421
IBI/HRV	Infrastructure	MIN_bpm	Curb	-2.9652	0.0117
IBI/HRV	Infrastructure	MIN_bpm	Group Var	0.2226	0.0009
IBI/HRV	Infrastructure	MIN_LF_HF_ratio	Building	-0.0402	0.0008
IBI/HRV	Infrastructure	MIN_LF_HF_ratio	Curb	0.0346	0.0252
IBI/HRV	Infrastructure	MIN_LF_HF_ratio	Group Var	0.149	0.0016
IBI/HRV	Infrastructure	MIN_LF_HF_ratio	Pole	-0.026	0.0254
IBI/HRV	Infrastructure	MIN_pNN50	Group Var	0.0818	0.0092
IBI/HRV	Infrastructure	STD_bpm	Curb	1.1302	0.0178
IBI/HRV	Infrastructure	STD_bpm	Group Var	0.1303	0.0027
IBI/HRV	Infrastructure	STD_bpm	Utility_Pole	-0.6179	0.0332
IBI/HRV	Infrastructure	STD_pNN50	Group Var	0.0718	0.0142
IBI/HRV	Infrastructure	STD_RMSSD	Group Var	0.0564	0.0281
IBI/HRV	NOISE	MAX_bpm	Group Var	0.244	0.0002
IBI/HRV	NOISE	MAX_bpm	NOISE_max	2.5523	0.0044
IBI/HRV	NOISE	MAX_bpm	NOISE_std	-2.0941	0.0285
IBI/HRV	NOISE	MAX_LF_HF_ratio	NOISE_max	0.0686	0.009
IBI/HRV	NOISE	MAX_pNN50	Group Var	0.099	0.0062
IBI/HRV	NOISE	MAX_pNN50	NOISE_max	0.0409	0.0006
IBI/HRV	NOISE	MAX_RMSSD	Group Var	0.0882	0.0061
IBI/HRV	NOISE	MAX_RMSSD	NOISE_max	7.8992	0.0001
IBI/HRV	NOISE	MEAN_bpm	Group Var	0.2934	0.0003
IBI/HRV	NOISE	MEAN_bpm	NOISE_std	-2.1996	0.0016
IBI/HRV	NOISE	MEAN_LF_HF_ratio	Group Var	0.0737	0.0321
IBI/HRV	NOISE	MEAN_LF_HF_ratio	NOISE_std	0.0264	0.0345
IBI/HRV	NOISE	MEAN_pNN50	Group Var	0.0897	0.0114
IBI/HRV	NOISE	MEAN_pNN50	NOISE_std	-0.0202	0.027
IBI/HRV	NOISE	MEAN_RMSSD	Group Var	0.1031	0.0091
IBI/HRV	NOISE	MIN_bpm	Group Var	0.2083	0.0012
IBI/HRV	NOISE	MIN_bpm	NOISE_max	-1.7214	0.0289
IBI/HRV	NOISE	MIN_LF_HF_ratio	Group Var	0.1372	0.0019
IBI/HRV	NOISE	MIN_LF_HF_ratio	NOISE_std	0.0277	0.0119
IBI/HRV	NOISE	MIN_pNN50	Group Var	0.081	0.009
IBI/HRV	NOISE	MIN_pNN50	NOISE_max	-0.0285	0.0022
IBI/HRV	NOISE	MIN_RMSSD	Group Var	0.0949	0.0062
IBI/HRV	NOISE	MIN_RMSSD	NOISE_max	-3.0153	0.0106
IBI/HRV	NOISE	STD_bpm	Group Var	0.1258	0.0036
IBI/HRV	NOISE	STD_pNN50	Group Var	0.0692	0.016
IBI/HRV	NOISE	STD_pNN50	NOISE_max	0.017	0.0005
IBI/HRV	NOISE	STD_RMSSD	Group Var	0.0525	0.0375
IBI/HRV	NOISE	STD_RMSSD	NOISE_max	2.3792	0.0005
IBI/HRV	POI	MAX_bpm	Group Var	0.2543	0.0001
IBI/HRV	POI	MAX_LF_HF_ratio	Group Var	0.0551	0.0331
IBI/HRV	POI	MAX_pNN50	Group Var	0.1089	0.0045
IBI/HRV	POI	MAX_RMSSD	Group Var	0.0994	0.0035
IBI/HRV	POI	MEAN_bpm	Group Var	0.3065	0.0002
IBI/HRV	POI	MEAN_LF_HF_ratio	Group Var	0.1234	0.0302
IBI/HRV	POI	MEAN_pNN50	Group Var	0.0888	0.0124
IBI/HRV	POI	MEAN_RMSSD	Group Var	0.0958	0.0128
IBI/HRV	POI	MIN_bpm	Group Var	0.219	0.0009
IBI/HRV	POI	MIN_bpm	poi_amenity	-1.9472	0.0128
IBI/HRV	POI	MIN_LF_HF_ratio	Group Var	0.1549	0.0023
IBI/HRV	POI	MIN_pNN50	Group Var	0.0908	0.0075
IBI/HRV	POI	STD_bpm	Group Var	0.1211	0.0033
IBI/HRV	POI	STD_bpm	poi_amenity	0.7005	0.0252
IBI/HRV	POI	STD_LF_HF_ratio	Group Var	0.0494	0.0481
IBI/HRV	POI	STD_pNN50	Group Var	0.085	0.0102
IBI/HRV	POI	STD_RMSSD	Group Var	0.0638	0.0223
IBI/HRV	Temperature	MAX_bpm	Group Var	0.2525	0.0001
IBI/HRV	Temperature	MAX_LF_HF_ratio	Group Var	0.0459	0.0477
IBI/HRV	Temperature	MAX_pNN50	Group Var	0.1053	0.005
IBI/HRV	Temperature	MAX_RMSSD	Group Var	0.0885	0.0053
IBI/HRV	Temperature	MEAN_bpm	Group Var	0.3141	0.0002
IBI/HRV	Temperature	MEAN_LF_HF_ratio	Group Var	0.0695	0.0378
IBI/HRV	Temperature	MEAN_pNN50	Group Var	0.0815	0.0161
IBI/HRV	Temperature	MEAN_RMSSD	Group Var	0.0866	0.0182
IBI/HRV	Temperature	MIN_bpm	Group Var	0.2123	0.0009
IBI/HRV	Temperature	MIN_LF_HF_ratio	Group Var	0.126	0.0025
IBI/HRV	Temperature	MIN_pNN50	Group Var	0.0736	0.013
IBI/HRV	Temperature	MIN_RMSSD	Group Var	0.0847	0.0105
IBI/HRV	Temperature	STD_bpm	Group Var	0.11	0.0054
IBI/HRV	Temperature	STD_pNN50	Group Var	0.0774	0.0104
IBI/HRV	Temperature	STD_RMSSD	Group Var	0.0543	0.0304
IBI/HRV	Visuals-Mapillary	MAX_bpm	Group Var	0.2247	0.0002
IBI/HRV	Visuals-Mapillary	MAX_bpm	Person	2.1648	0.0111
IBI/HRV	Visuals-Mapillary	MAX_LF_HF_ratio	Group Var	0.0467	0.0407
IBI/HRV	Visuals-Mapillary	MAX_pNN50	Group Var	0.1091	0.0041
IBI/HRV	Visuals-Mapillary	MAX_pNN50	Person	0.0296	0.0099
IBI/HRV	Visuals-Mapillary	MAX_RMSSD	Group Var	0.09	0.0042
IBI/HRV	Visuals-Mapillary	MAX_RMSSD	Person	5.3619	0.0068
IBI/HRV	Visuals-Mapillary	MEAN_bpm	Car	2.1891	0.0012
IBI/HRV	Visuals-Mapillary	MEAN_bpm	Group Var	0.2941	0.0003
IBI/HRV	Visuals-Mapillary	MEAN_LF_HF_ratio	Car	-0.0256	0.0375
IBI/HRV	Visuals-Mapillary	MEAN_LF_HF_ratio	Group Var	0.0718	0.0323
IBI/HRV	Visuals-Mapillary	MEAN_RMSSD	Group Var	0.1021	0.0091
IBI/HRV	Visuals-Mapillary	MIN_bpm	Group Var	0.2135	0.0009
IBI/HRV	Visuals-Mapillary	MIN_LF_HF_ratio	Car	-0.0222	0.038
IBI/HRV	Visuals-Mapillary	MIN_LF_HF_ratio	Group Var	0.1266	0.0023
IBI/HRV	Visuals-Mapillary	MIN_LF_HF_ratio	Person	-0.0202	0.0413
IBI/HRV	Visuals-Mapillary	MIN_pNN50	Group Var	0.0806	0.0088
IBI/HRV	Visuals-Mapillary	STD_bpm	Car	0.6937	0.0368
IBI/HRV	Visuals-Mapillary	STD_bpm	Group Var	0.1156	0.0039
IBI/HRV	Visuals-Mapillary	STD_pNN50	Group Var	0.0742	0.0118
IBI/HRV	Visuals-Mapillary	STD_pNN50	Person	0.0124	0.0074
IBI/HRV	Visuals-Mapillary	STD_RMSSD	Group Var	0.0524	0.0301
IBI/HRV	Visuals-Mapillary	STD_RMSSD	Person	1.846	0.0047
Skin Temperature	Infrastructure	MAX_Values	Utility_Pole	0.0883	0.0334
Skin Temperature	Infrastructure	MIN_Values	Curb	-0.1936	0.0198
Skin Temperature	Infrastructure	MIN_Values	Manhole	0.1919	0.0032
Skin Temperature	Infrastructure	STD_Values	Curb	0.0446	0.0053
Skin Temperature	Infrastructure	STD_Values	Group Var	0.1169	0.0008
Skin Temperature	Infrastructure	STD_Values	Manhole	-0.0308	0.0152
Skin Temperature	NOISE	STD_Values	Group Var	0.1123	0.0014
Skin Temperature	POI	MIN_Values	public_services	-0.2074	0.0161
Skin Temperature	POI	STD_Values	Group Var	0.1212	0.0008
Skin Temperature	Temperature	MAX_Values	Temp_mean	0.4064	0.0284
Skin Temperature	Temperature	STD_Values	Group Var	0.1157	0.0008
Skin Temperature	Visuals-Mapillary	MAX_Values	Fence	0.1469	0.0073
Skin Temperature	Visuals-Mapillary	STD_Values	Fence	0.0375	0.0036
Skin Temperature	Visuals-Mapillary	STD_Values	Group Var	0.1056	0.0013

Table S4. Standardized Coefficients (β) and p-values from Mixed-Effects Models Examining the Effects of Environmental Stressors on Physiological Responses During Urban Walking (H2)

Note: Results from linear mixed-effects models (random intercept for participant ID) examining trip-level associations between environmental conditions and physiological indicators of electrodermal activity (EDA), heart-rate variability (HRV), and skin temperature. Analyses included 2,207 walking trips from 90 participants. Predictors were grouped into five blocks: Infrastructure (e.g., curbs, poles, manholes), Noise (mean, max, SD), Temperature (mean, max), Visuals (Mapillary features such as cars or people), and Points of Interest (OSM). Environmental variables were aggregated within a 25 m buffer along each trip and standardized (z-scores). Thus, each β represents the mean change in the outcome associated with a one-SD increase in the predictor.

No additional sociodemographic covariates were included; individual differences were modeled via the random intercept. Only associations with $p < 0.05$ are displayed. Positive β values indicate higher physiological activation, while negative βs indicate reduced activation.

System	Variable	Statistic	p-value
**EDA**	**STD amplitude**	**8.094**	**0.0441**
EDA	STD rise time npoints ratio	4.234	0.2372
**EDA**	**MAX amplitude mean**	**9.095**	**0.0281**
EDA	MAX rise time npoints ratio	7.35	0.0615
**EDA**	**MEAN amplitude mean**	**12.238**	**0.0066**
**EDA**	**Mean rise time npoints ratio**	**14.263**	**0.0026**
**EDA**	**MIN amplitude mean**	**18.842**	**0.0003**
EDA	MIN rise time npoints ratio	3.439	0.3288
IBI/HRV	STD bpm mean	3.514	0.319
IBI/HRV	STD LF HF ratio	1.726	0.6311
IBI/HRV	STD pNN50	0.179	0.9809
IBI/HRV	STD RMSSD	2.369	0.4995
IBI/HRV	MAX bpm mean	1.457	0.6923
IBI/HRV	MAX LF HF ratio	4.294	0.2314
IBI/HRV	MAX pNN50	3.282	0.3502
IBI/HRV	MAX RMSSD	3.387	0.3357
IBI/HRV	MEAN bpm mean	4.464	0.2155
IBI/HRV	MEAN LF HF ratio	6.714	0.0816
IBI/HRV	MEAN pNN50	5.287	0.1519
IBI/HRV	MEAN RMSSD	5.957	0.1137
IBI/HRV	MIN bpm mean	6.617	0.0852
IBI/HRV	MIN LF HF ratio	1.978	0.577
IBI/HRV	MIN pNN50	2.657	0.4476
IBI/HRV	MIN RMSSD	5.111	0.1638
Perception	Agitated - Calm	1.966	0.5794
Perception	Tired - Awake	2.339	0.5051
**Perception**	**Trip Feelings Environment**	**9.767**	**0.0207**
Perception	Unwell - Well	5.054	0.1679
**Skin Temperature**	**STD Values mean**	**17.765**	**0.0005**
Skin Temperature	MAX Values mean	1.118	0.7727
Skin Temperature	MEAN Values mean	6.38	0.0945
**Skin Temperature**	**MIN Values mean**	**11.551**	**0.0091**

Table S5. Differences in Subjective and Physiological Responses Across Environmental Clusters Identified by K-means Analysis (H3)

Note:
Environmental clusters were derived using K-means clustering applied to standardized trip-level environmental indicators (NDVI, noise exposure, built infrastructure, and points of interest). Four distinct clusters were identified, representing gradients from greener and quieter routes (Clusters 0–1) to dense, noisy, and infrastructure-rich environments (Cluster 3).
The table reports Kruskal–Wallis test statistics (χ^2) and p-values comparing both subjective perceptions (e.g., Trip Feeling Environment, Calmness, Well-being) and physiological responses (e.g., EDA amplitude, HRV, and skin temperature) across clusters.
Results are exploratory and describe how combined environmental typologies are associated with variations in psychophysiological states during walking. Positive or higher values indicate greater activation or more favorable perceptions depending on the variable. Statistical significance was set at α = 0.05.
Clusters were not used as predictors in mixed-effects models because the goal of H3 was to explore multivariate environmental typologies, not to estimate directional effects of cluster membership.

INDIVID	Walk trips	Mean Time Per Trip (minutes)	Mean Distance per Trip (meters)	INDIVID	Walk trips	Mean Time Per Trip (minutes)	Mean Distance per Trip (meters)
MMM29001	17	14.5	1145.7	MMM34001	17	6.5	517.6
MMM29101	21	14.0	1059.0	MMM34101	18	8.0	670.1
MMM29201	42	6.0	540.7	MMM34201	41	10.5	694.9
MMM29301	13	19.5	2333.3	MMM34301	8	7.0	703.2
MMM29701	45	7.8	688.4	MMM34401	63	14.2	1125.3
MMM29801	26	6.2	605.2	MMM34501	4	1.5	189.3
MMM29901	79	7.2	620.8	MMM34601	35	7.4	661.7
MMM30001	54	10.4	699.1	MMM34701	13	6.3	585.1
MMM30101	13	11.6	1297.3	MMM34801	36	5.0	365.3
MMM30201	45	5.1	500.4	MMM34901	58	14.3	1132.0
MMM30301	24	10.7	736.9	MMM35001	23	11.2	869.2
MMM30401	25	7.2	520.9	MMM35101	11	9.1	604.1
MMM30501	23	7.1	765.7	MMM35201	23	16.1	1319.8
MMM30601	32	12.0	966.5	MMM35301	7	6.6	598.6
MMM30701	17	5.5	484.0	MMM35401	4	2.5	173.2
MMM30801	32	7.4	579.6	MMM35501	40	14.3	1486.1
MMM30901	1	16.5	1217.1	MMM35601	8	5.9	747.2
MMM31001	21	11.0	929.4	MMM35701	14	10.1	862.9
MMM31101	27	17.7	1621.3	MMM35801	31	10.0	852.2
MMM31201	4	8.2	722.1	MMM36201	46	10.8	921.7
MMM31401	39	14.4	1332.8	MMM36401	2	5.5	325.8
MMM31501	52	7.1	521.1	MMM36501	50	7.8	572.9
MMM31601	114	7.7	648.6	MMM36701	6	6.6	425.6
MMM31701	1	7.3	823.3	MMM36801	22	8.2	563.0
MMM31801	14	8.9	618.7	MMM36901	24	14.0	793.7
MMM31901	8	8.6	766.9	MMM37001	78	8.2	531.4
MMM32001	17	9.5	707.5	MMM37101	10	9.0	761.8
MMM32101	28	11.2	933.7	MMM37201	43	6.1	488.9
MMM32201	16	8.4	608.2	MMM37301	14	14.9	993.4
MMM32301	21	5.5	486.7	MMM37401	5	3.4	434.3
MMM32401	27	8.2	768.9	MMM37501	83	7.4	642.7
MMM32501	19	8.4	635.2	MMM37601	20	9.0	695.9
MMM32601	13	8.5	622.1	MMM37701	15	6.8	648.3
MMM32701	3	12.4	607.4	MMM37901	22	7.1	644.9
MMM32901	36	6.9	567.9	MMM38001	4	7.1	589.1
MMM33001	51	7.6	644.2	MMM38101	53	6.7	561.8
MMM33101	59	21.2	1638.2	MMM38201	1	5.0	404.5
MMM33201	7	11.6	881.2	MMM38301	12	7.7	600.1
MMM33301	13	2.8	309.7	MMM38401	13	7.2	458.6
MMM33501	16	12.3	1099.9	MMM38501	4	15.5	933.6
MMM33601	32	7.9	682.2	MMM38601	38	4.9	402.1
MMM33701	5	2.8	250.4	MMM35901	0	0	0
MMM33801	4	2.4	170.9	MMM36301	0	0	0
MMM33901	32	4.7	484.5	MMM36601	0	0	0
MMM33401	0	0	0	MMM37801	0	0	0

Table S6. Summary of Walking Trips per Participant

Note: Each row represents one participant (N = 90). “Walk trips” = number of validated walking segments; “Mean Time per Trip” = average trip duration (minutes); “Mean Distance per Trip” = average trip length (meters). Participants with zero valid trips (n = 5) are shown for completeness but excluded from analyses.
